# Alternative splicing during mammalian organ development

**DOI:** 10.1038/s41588-021-00851-w

**Published:** 2021-05-03

**Authors:** Pavel V. Mazin, Philipp Khaitovich, Margarida Cardoso-Moreira, Henrik Kaessmann

**Affiliations:** 1 V. Zelman Center for Neurobiology and Brain Restoration, Skolkovo Institute of Science and Technology, Moscow, Russia; 2 Center for Molecular Biology of Heidelberg University (ZMBH), DKFZ-ZMBH Alliance, Heidelberg, Germany; 3 Present Address: Evolutionary Developmental Biology Laboratory, The Francis Crick Institute, London, UK

**Keywords:** Gene expression, Organogenesis

## Abstract

Alternative splicing (AS) is pervasive in mammalian genomes, yet cross-species comparisons have been largely restricted to adult tissues and the functionality of most AS events remains unclear. We assessed AS patterns across pre- and postnatal development of seven organs in six mammals and a bird. Our analyses revealed that developmentally dynamic AS events, which are especially prevalent in the brain, are substantially more conserved than nondynamic ones. Cassette exons with increasing inclusion frequencies during development show the strongest signals of conserved and regulated AS. Newly emerged cassette exons are typically incorporated late in testis development, but those retained during evolution are predominantly brain specific. Our work suggests that an intricate interplay of programs controlling gene expression levels and AS is fundamental to organ development, especially for the brain and heart. In these regulatory networks, AS affords substantial functional diversification of genes through the generation of tissue- and time-specific isoforms from broadly expressed genes.

## Abstract (web summary)

Analysis of RNA-seq datasets from seven organs across seven species generates an alternative splicing (AS) atlas and shows that AS events provide functional gene diversification through generation of tissue- and time-specific transcript isoforms.

## Main

AS is a process in which splice sites are differentially selected within pre-messenger RNAs to generate distinct RNA and protein isoforms^1^. AS is pervasive in mammals, affecting most multi-exonic genes and accounting for an immense isoform diversity^2^. Many regulatory aspects of AS networks and functions have been uncovered^2^. However, the functional relevance of most alternative isoforms remains unknown^2^. Genome-scale investigations reported that only small proportions of AS events show evolutionary conservation or have other broad-scale functional support^3–10^. Therefore, it was suggested that much of the isoform diversity arising from AS represents transcriptional noise^4,6–10^, although this interpretation has been intensely debated^8,9,11,12^.

Cross-species comparisons provide a powerful framework to globally assess the functionality, regulatory mechanisms and evolutionary dynamics of AS; for example, they uncovered the overall rapid turnover of AS events during evolution and identified ancestral mammalian splicing regulators and associated binding motifs^5,13^. However, this work has been restricted to adult tissues (with exceptions^2,14–17^), even though AS may play essential roles during organ development^2,17^.

To fill this critical gap, we generated developmental AS atlases of seven organs across seven species (https://apps.kaessmannlab.org/alternative-splicing), based on an extensive RNA-sequencing (RNA-seq) dataset^18^. Integrated comparative analyses of these atlases uncovered the evolutionary dynamics and functional relevance of AS across mammalian organ development.

### Developmental AS atlases

**Fig. 1:**
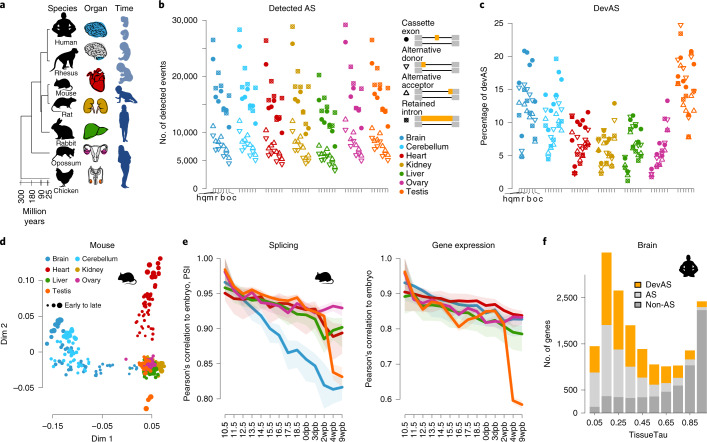
Developmental AS atlases. **a**, Schematic representation of the underlying RNA-seq dataset (1,890 libraries). Full details are given in Extended Data Fig. 1. **b**, Numbers of detected AS events for the four major classes across organs and species. Species appear in the same order and are denoted by letters: human (h), macaque (q), mouse (m), rat (r), rabbit (b), opossum (o) and chicken (c). **c**, Percentages of devAS events (among all detected AS events shown in **b**) for the four major AS classes across organs and species (*x* axis as shown in **b**). **d**, MDS plots for mouse samples based on pairwise distances (1 – *r*, Pearson’s correlation coefficient) between PSI values of all alternatively spliced cassette exons. **e**, Pearson’s correlation coefficient (*r*) of cassette exon inclusion frequencies (PSIs; left) and gene expression levels (log_2_-transformed reads per kilobase per million mapped reads (RPKM)) (right), between the earliest (10.5 d post-conception) and all successive developmental stages for different mouse organs. The lines depict the median and the shadings the 95% confidence intervals (CIs) based on bootstrapping analysis (1,000 replicates, with all possible pairs of samples between given and earliest stage considered). **f**, Numbers of genes with different tissue specificity (TissueTau) and with devAS (dPSI > 0.2), nondynamic AS (AS) or no alternatively spliced cassette exons (non-AS) for human brain. Organ and species icons (except human) are from a previous study^18^.

**Extended Data Fig. 1:**
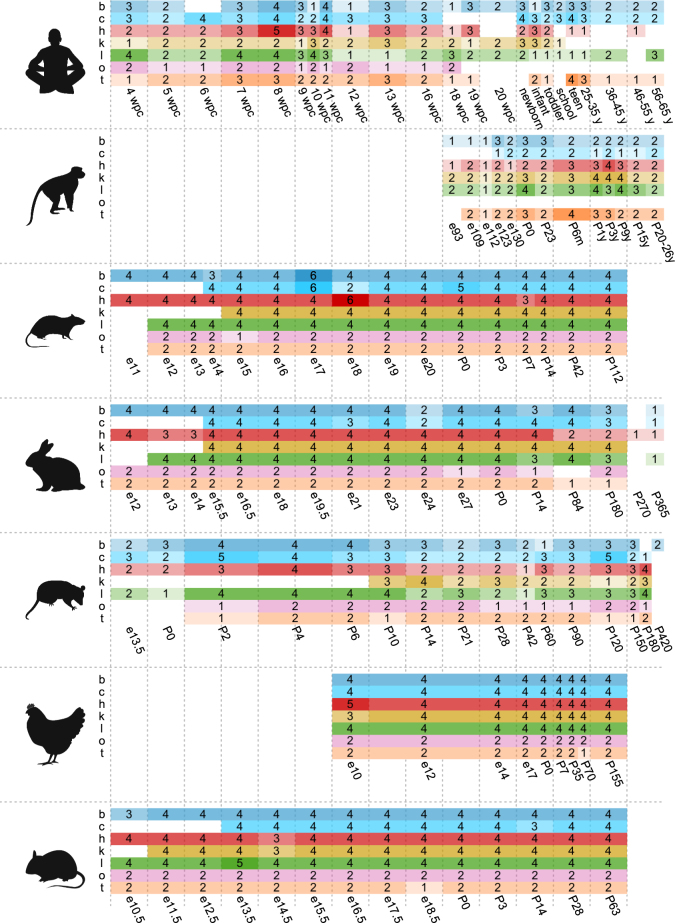
Overview of the dataset. Numbers of replicates available for each species, organ, and developmental stage. The developmental stages are aligned across species using mouse as a reference^18^ (dotted vertical lines). The organs are denoted by colors and letters (brain, cerebellum, heart, kidney, liver, ovary, and testis). Species icons (except human) are from a previous study^18^.

To study the evolution of developmental AS, we leveraged an RNA-seq dataset comprising 1,890 libraries spanning the development of 7 organs (forebrain/cerebrum, hindbrain/cerebellum, heart, kidney, liver, ovary and testis) from early organogenesis (mid-organogenesis for the heart) to adulthood across six mammals (human, rhesus macaque, mouse, rat, rabbit and opossum) and a bird (chicken)^18^ (Fig. 1a; exceptions and details in Extended Data Fig. 1). We performed detailed de novo annotations of transcribed regions for all species, including precise mapping of splice sites (Supplementary Data 1–7). This de novo annotation prevents biases in downstream analyses due to genome annotation quality differences between species, and detects novel splice variants^15,19,20^ (Supplementary Fig. 1 and Methods). We defined gene segments as the sequences between two adjacent splice sites (Supplementary Fig. 2). Alternative segments (that is, segments not included in all transcript isoforms of genes) were classified into the four major AS classes: alternative cassette exons, alternative donor segments, alternative acceptor segments and intron retention events (Fig. 1b). Inclusion frequencies (percentage spliced-in (PSI)) for each alternative segment and 1:1 orthologous exons across species (Supplementary Data 8) were determined using established procedures^15,19,20^ (Methods).

**Extended Data Fig. 2:**
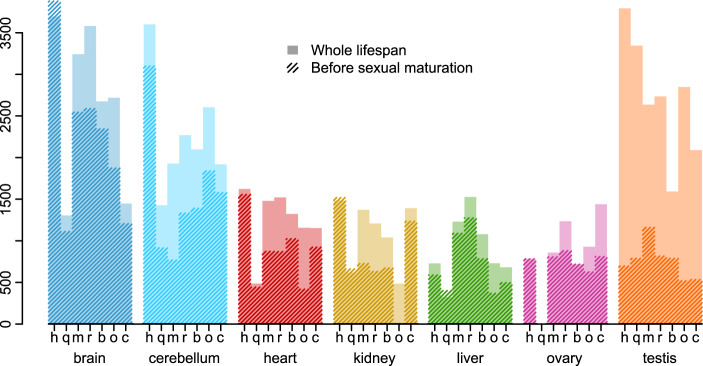
Influence of including developmental stages from sexually mature individuals on the detection of cassette exons with devAS. Cassette exons with devAS detected in data restricted to organ development before sexual maturation (dark colors) and in data covering the whole developmental span (that is, including sexual maturation/adult stages; light colors). Species are denoted by letters: human (h), macaque (q), mouse (m), rat (r), rabbit (b), opossum (o), and chicken (c).

We identified thousands of mostly coding AS events for each of the four classes in each organ from the seven species (Fig. 1b, Supplementary Table 1 and Methods). Cassette exons and intron retention are the most frequent classes, as previously observed^21^. Within each class and species, the number of detected AS events is comparable across organs^21^ (Fig. 1b and Supplementary Fig. 3). By contrast, among AS events with significant changes of inclusion frequencies through time (termed ‘developmentally dynamic AS’ (devAS); Methods), brain tissues and testis stand out, with substantially larger numbers and proportions of devAS events than the other organs (Fig. 1c, Supplementary Fig. 3 and Supplementary Data 9). The testis undergoes a fundamental change in cellular composition during sexual maturation, with spermatogenic cells becoming predominant^22,23^. These cell types have pervasive open chromatin that facilitates transcriptional noise of various types, including AS^23–26^. Consistently, we demonstrate that the sexually mature testis drives the high level of devAS for all classes in the testis (Fig. 1c); that is, the excess of devAS in the testis disappears when considering developmental stages before sexual maturation (Extended Data Fig. 2). Therefore, the testis’ devAS signal probably reflects a cell compositional rather than a developmental AS program switch. By contrast, the brain’s excess of devAS events remains when restricting the analysis to pre-sexual maturity stages. The pronounced devAS signal in the brain may thus reflect the particular importance of AS for this organ’s development.

We investigated how differences in developmental sampling across species explain differences in the numbers of detected AS and devAS events (Fig. 1b,c and Supplementary Fig. 3) using subsampling analyses. These analyses revealed that the number of AS and devAS events in macaques, for which we lack early prenatal data (Extended Data Fig. 1), become similar to those in humans when sampling schemes are matched (Supplementary Fig. 3). Altogether, our subsampling analyses suggest that the main difference between species is the larger number of detected AS events in the primates than in the other species (Supplementary Fig. 3), consistent with AS analyses in adults^3^. However, the fact that numbers of devAS events are not larger in primates suggests that the primate excess in detected AS events reflects nearly neutral (mildly deleterious) transcriptional noise in primates associated with their lower effective population sizes (that is, weaker purifying selection)^24,27^.

Given the large numbers of devAS cassette exons and the strong previous focus on this AS class^3,5,17,21^, we focused all subsequent analyses on cassette exons.

### AS has disproportionate roles in brain and heart development

To explore global AS relationships among samples, we performed multidimensional scaling (MDS) analyses in each species (Methods). Samples cluster by organ and, for the heart and especially the brain tissues, samples are ordered by developmental stage (Fig. 1d and Supplementary Fig. 4). This clustering suggests AS programs that steadily diverge during development, especially in the brain and heart. Indeed, comparisons of developmental AS states relative to the earliest embryonic stage based on PSI correlations revealed a progressive divergence of AS patterns during development, but with a substantially higher rate for the brain (Fig. 1e and Supplementary Fig. 4). This contrasts with gene expression levels, where the brain is not an outlier (Fig. 1e and Supplementary Fig. 4). The sudden drop in the correlation of AS in the testis upon sexual maturation is also seen for gene expression levels and is probably due to the emergence of major spermatogenic cell populations with widespread transcriptional noise^23–26^ (see previous section). Therefore, this drop does not represent a functional AS program switch, but rather reflects a lack of connection between AS patterns before and after sexual maturation owing to a fundamental change in cellular composition. Together with the larger proportion of devAS events in brain tissues (Fig. 1c), these analyses indicate that AS programs play a disproportionate role in brain and, to a lesser extent, heart development. However, the heart’s less pronounced devAS patterns might be due to the heart being more developed than the brain (and the other organs) at our earliest sampled stage.

**Extended Data Fig. 3:**
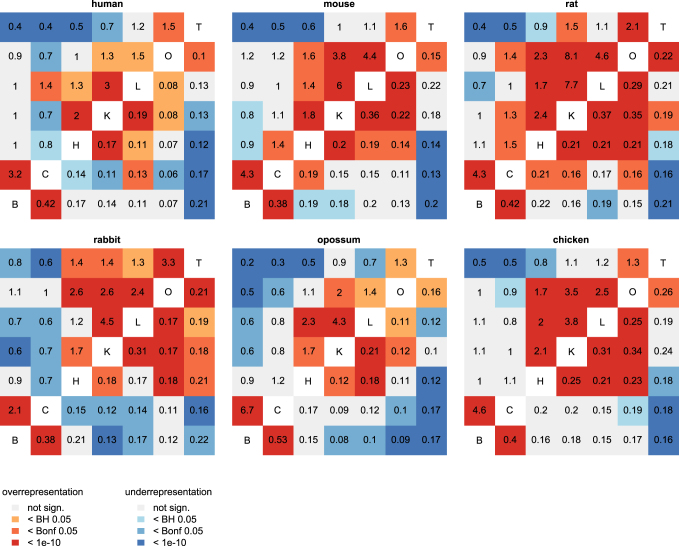
Organ overlap patterns for cassette exons with devAS in multiple organs. Heatmaps show overlaps of cassette exons for organ pairs in six species (macaque was excluded from this analysis due to the shorter developmental span covered): brain (B), cerebellum (C), heart (H), kidney (K), liver (L), ovary (O), testis (T). Upper-left triangles show odds ratios, lower-right triangles show intersect to union ratios. Colors indicate *P*-values (Fisher’s exact tests) and levels of potential over-/underrepresentation (see inset legends; ‘BH’ and ‘Bonf’ stand for Benjamini Hochberg and Bonferroni correction, respectively).

We then investigated the organ specificity of devAS. We find that most devAS events (64–84% depending on the species) are specific to one organ, consistent with observations in adults^12^. Notably, for cassette exons with devAS in more than one organ, specific organ pairs are observed more frequently than others. As expected, forebrain/cerebrum and hindbrain/cerebellum form the most frequent pair (*P* *<* 10^−34^, odds ratio (OR) = 2.1–6.7, Fisher’s exact test), but surprisingly, kidney and liver pairs are also overrepresented (*P* *<* 10^−29^, OR = 3.0–7.7, Fisher’s exact test; Extended Data Fig. 3). These results suggest that the regulation of AS programs is shared and potentially coordinated across organs. Notably, most devAS events (and AS events in general) occur in genes with broad spatial expression profiles (Fig. 1f), a result consistent across genes with varying exon numbers (Supplementary Fig. 5). For example, 75% of devAS events in the human brain occur in genes with broad spatial expression (tissue specificity <0.5; Fig. 1f). These results suggest that devAS may allow for organ-specific developmental functions in ubiquitously expressed genes.

### High conservation of developmentally dynamic AS

**Fig. 2:**
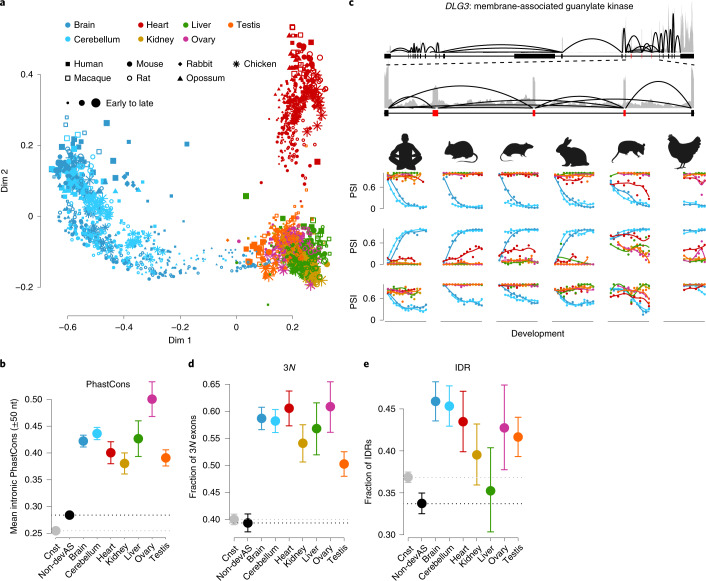
Conservation of devAS events. **a**, MDS plots for all 1,890 samples, based on pairwise distances (1 − *r*, Pearson’s correlation coefficient) between PSI values of 1,441 orthologous cassette exons (that is, exons that are alternatively spliced in all seven species). **b**, Mean primate sequence conservation (PhastCons^51^) scores of intronic sequences flanking human cassette exons (that is, 50 nt upstream and downstream) in the different organs. Values for constitutive (cnst, in gray) and nondevelopmentally dynamic exons (non-devAS, black) are shown for comparison. Data are presented as the mean values ± 2 × s.e.m. **c**, Examples of conserved devAS events: three cassette exons in *DLG3*. Top: total read coverage for all exons of the mouse *DLG3* gene (reads summed across all mouse samples); zoomed region is shown below, with cassette exons in red. Bottom: developmental PSI trajectories across species-matched developmental stages^18^ of the three cassette exons in six species (chicken: earlier developmental samples not available; macaque not shown due to the shorter temporal range of the samples). **d**, Proportions of human devAS exons with length divisible by three across organs. **e**, Proportions of human devAS exons that encode an intrinsically disordered region across organs. In **d** and **e**, the dots represent the mean values and the error bars the 95% CIs, based on binomial distributions. The species icons (except human) are from a previous study^18^.

Next, we characterized the evolutionary conservation of AS across organs. We found that most (∼65–81%) 1:1 orthologous cassette exons alternatively spliced across all species show devAS in at least one organ in all species, and nearly all (97%) show devAS in at least one species. By contrast, only ∼31–38% of exons alternatively spliced in each species display devAS in at least one organ. An MDS analysis of the 1,441 orthologous cassette exons (Fig. 2a) mirrors the species-specific MDS analyses (Fig. 1d and Supplementary Fig. 4). Samples cluster by organ and, for heart and brain tissues, samples are ordered by developmental stage (Fig. 2a). The organ-dominated clustering suggests that organs have conserved devAS signatures. The strong conservation of devAS is further supported by analyses of the intronic sequences flanking cassette exons (that is, sequences potentially harboring AS *cis*-regulatory sequences^28^), which reveal significantly higher conservation scores for devAS than non-devAS events (Fig. 2b; *P* < 10^−10^ for all organs, Mann–Whitney *U*-test), consistent with results from mouse cortex development^16^. Examples of highly conserved devAS events in the brain are the three cassette exons of *DLG3*, which encodes a synapse-associated protein implicated in learning disability^29,30^ (Fig. 2c).

The conservation of devAS suggests that the repertoires of devAS are enriched for actively regulated and thus functional AS events. Indeed, we find that the proportion of exons that preserve the reading frame is substantially higher among devAS than non-devAS events (Fig. 2d; *P* < 10^−10^ for all organs, proportion test), consistent with work from mouse cortex development^16^. Furthermore, devAS exons are significantly enriched in their coding potential for intrinsically disordered regions of proteins—potential regulators of protein interaction networks^31^—compared with other alternatively spliced exons (Fig. 2e; *P* < 0.005 for all organs except liver, proportion test).

### Early versus late development

**Fig. 3:**
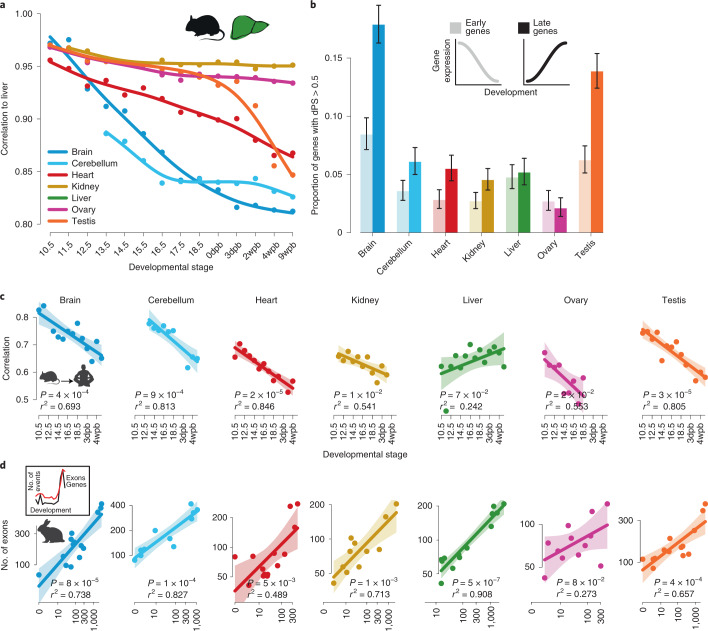
Early versus late development: interplay between gene expression and AS. **a**, Pearson’s correlation coefficients for comparisons of PSI values of cassette exons (*n* = 20,186) between mouse liver and each of the other organs across developmental stages. **b**, Proportion of exons with absolute amplitude of developmental change (dPSI) > 0.5 among early and late expressed genes^18^. Error bars: 95% CIs, based on binomial distributions. **c**, Pearson’s correlation coefficients for comparisons of PSI values of orthologous cassette exons between mice and humans across development in the different organs (only exons with AS in both species that are devAS in at least one are considered). **d**, Correlation between numbers of genes (*x* axis) and cassette exons with devAS (*y* axis) that changed in expression and PSI (difference in PSI > 0.2), respectively, between consecutive developmental stages in rabbit (Methods). Inset (to the left) shows numbers of cassette exons (red) and genes (black) with PSI and expression change, respectively, between consecutive developmental stages in rabbit brain across development. In **c** and **d**, the linear fit and 95% CIs are shown by the line and shading, respectively. Unadjusted analysis of variance *P* values and *r*^2^ for fits are shown in each panel. Organ and species icons (except human) are from a previous study^18^.

Protein-coding gene expression levels are most similar across organs at the earliest developmental stages and then gradually diverge into distinct developmental programs^18^. We find that AS programs parallel this temporal diversification of expression patterns by progressively diverging between organs as development advances, especially for the brain (Fig. 3a and Supplementary Fig. 6). AS patterns in the testis show a sharp increase in divergence from those in other organs on sexual maturation, consistent with our previous observations (Fig. 1e, Extended Data Fig. 2 and Supplementary Fig. 4) and patterns reported for gene expression levels^18^. Our analyses also show that genes predominantly expressed early in development show lower rates of devAS than late expressed genes, especially in the brain and testis (Fig. 3b). These observations suggest that devAS plays an important role in organ differentiation.

We further uncovered that early devAS events have been more strongly preserved during evolution than late devAS events (Fig. 3c and Supplementary Fig. 7). This is consistent with divergence patterns for both coding and noncoding gene expression^18,32^. Thus, despite lower rates of devAS in genes employed early in development (Fig. 3b), devAS has been subject to stronger selective constraints during this time, arguing for its importance during early organogenesis.

The rate of change of gene expression levels is not constant across development^18,32^. One major period of change is associated with the establishment of organ identity in early development and another with the transition to mature organ-specific functions^18,32^. We find that the rates of change of PSI for devAS and gene expression levels between consecutive stages are highly correlated during development in all species (Fig. 3d and Supplementary Fig. 8). This correlation implies that periods during development that show greater gene expression change (particularly the two periods described) also show larger devAS changes. However, we find that this occurs primarily through different sets of genes. For the two major periods of developmental change, only ∼10% (range: 0–55%) of genes showing significant changes in devAS also show significant gene expression changes. Despite this limited overlap, we still observe more genes changing by both mechanisms than expected by chance (in 30 out of 105 comparisons, Fisher’s exact test Benjamini–Hochberg-adjusted *P* < 0.05; Supplementary Table 2). Overall, our analyses indicate that development in general and key ontogenetic periods in particular are shaped by an interplay of programs controlling gene expression levels and AS.

### Exon usage across development

**Fig. 4:**
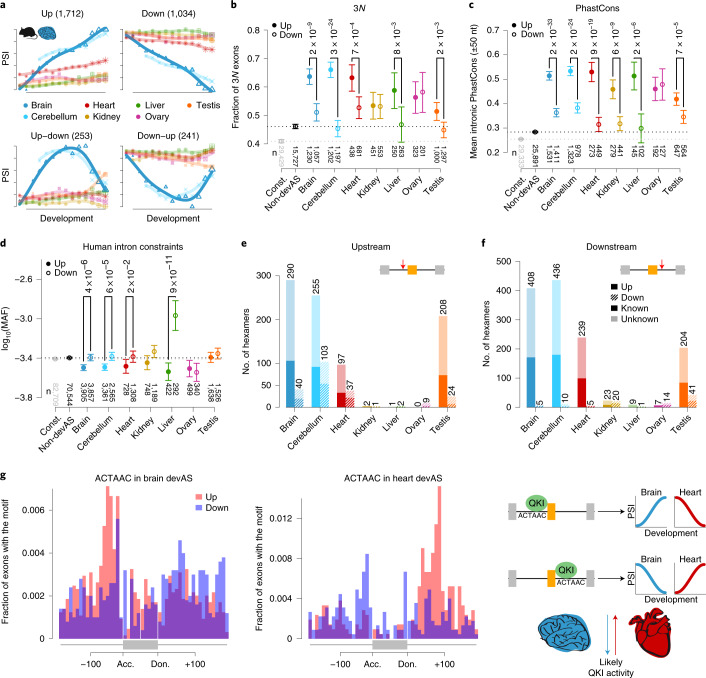
Developmental exon usage. **a**, Average PSI patterns for the four defined devAS classes in mouse brain. Numbers of cassette exons in each class are shown. **b**, Proportions (means and 95% CIs based on binomial distributions) of up (filled circles) and down (open circles) protein-coding segments with length divisible by three. Values for constitutive (cnst, in gray) and non-devAS (black) exons are shown for comparison. **c**, Mean primate sequence conservation (PhastCons^51^) scores of intronic sequences flanking human up/down exons (50 nt upstream/downstream). **d**, Mean (log_10_(MAF)) of human nonsingleton SNPs in intronic sequences flanking up/down cassette exons (50 nt upstream/downstream). In **c** and **d**, data are presented as mean values ± 2 × s.e.m. Two-sided binomial tests (**b**) and Mann–Whitney *U*-tests (**c**, **d**) were performed; unadjusted *P* values are shown (on top) for comparisons where *P* < 0.05. Bottom of **b**, **c** and **d**, respectively: numbers (*n*) of segments, exons and SNPs. **e**,**f**, Numbers of hexamers significantly enriched in upstream (**e**) or downstream (**f**) intronic sequences flanking devAS exons. Up and down exons: filled and hatched bars, respectively. Hexamers closely resembling known binding motifs of RNA-binding proteins from the CISBP–RNA database^52^ are indicated by brighter colors. **g**, Positional effect of QKI binding in brain and heart. Distribution of the ACTAAC hexamer in introns flanking brain (left) and heart (right) devAS exons. Data were combined across species (see Supplementary Fig. 13 for species-specific plots). Right panel: potential mechanism underlying opposing effects of QKI binding on targeted exons in the brain and heart. QKI binding acts as a repressor when binding upstream of a cassette exon and as an activator when binding downstream^35^. In the brain, the activity of QKI binding and/or co-regulators probably decreases over time (blue arrow), leading to increasing inclusion frequencies of exons with upstream QKI binding motifs during development (up pattern). In the heart, QKI binding/co-regulatory activities increase over time (red arrow), leading to decreasing inclusion frequencies of exons with upstream QKI binding motifs during development (PSI down pattern). Downstream motif localizations have inverse effects. acc., acceptor; don., donor. Organ and species icons are from a previous study^18^.

**Extended Data Fig. 4:**
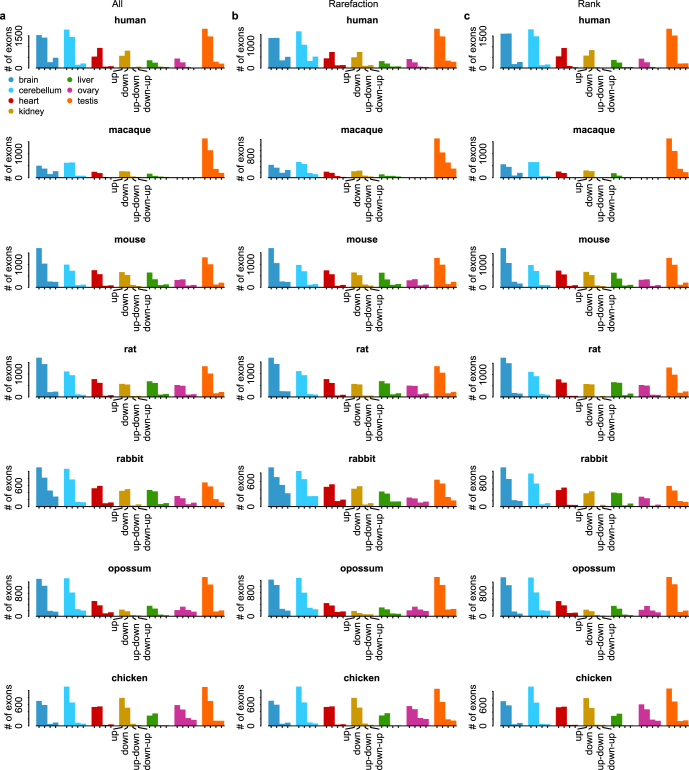
Frequencies of the four main developmental cassette exon usage patterns across organs and species. **a**, Numbers of devAS cassette exons with each of the four patterns are shown for all organs and species. Patterns are shown in the order indicated for kidney (that is, up, down, up-down and down-up). **b**, Same as (**a**) but samples were rarefied before the pattern analysis to make their distribution across development more even. For each species and organ, we split the whole developmental span (in a logarithmic scale of days post-conception, DPC) into 10 equally sized bins. Then we calculated the minimal number of samples per bin and randomly took as many (but not less than one) samples from each bin. **c**, Same as (**a**) but a rank of the developmental stage was used instead of the log(DPC) for the pattern analysis. The analyses in (**b**) and (**c**) were designed to test the dependence of the pattern analysis on the evenness of the distribution of samples across development.

To investigate the temporal patterns of devAS, we classified exons into four main patterns for each organ (Fig. 4a, Extended Data Fig. 4 and Supplementary Figs. 9 and 10): a progressive increase of the inclusion frequency during development (termed ‘up’), progressive decrease of the inclusion frequency (‘down’), increase followed by a decrease (‘up–down’) and decrease followed by an increase (‘down–up’). Notably, across species, most devAS exons (59–95%) show up or down patterns (23–58% and 24–58%, respectively; Fig. 4a, Extended Data Fig. 4 and Supplementary Fig. 10). We obtained similar results when enforcing regular sampling throughout development (Extended Data Fig. 4). Overall, the up pattern tends to be the most prevalent across organs and species, consistent with previous studies of mouse cortex^16^ and heart^14^ development (Fig. 4a and Extended Data Fig. 4). Furthermore, for 80% of cases showing up or down patterns in two organs, the direction of temporal change is the same. This concordance supports developmental AS regulation being coordinated between organs (consistent with the overrepresentation of organ pairs; Extended Data Fig. 3) and involving common regulators (below).

These specific temporal patterns result in major developmental divergences of AS between organs (Fig. 4a and Supplementary Fig. 10); that is, up and up–down patterns of cassette exons in specific organs are typically accompanied by low and nondevelopmentally dynamic inclusion frequencies of these exons in the other organs. Conversely, down and down–up patterns are typically accompanied by overall high/steady and nondynamic inclusion frequencies of these exons in the remaining organs. These spatiotemporal patterns are probably associated with early AS programs being very similar among organs and then progressively diverging during development (Fig. 3a).

The sequence conservation of flanking introns and the proportion of frame-preserving exons are significantly higher for up than for down cassette exons (Fig. 4b–d). These observations suggest that developmental inclusion increase is of greater functional relevance than decrease, which may more frequently correspond to AS noise. Consistently, we detected significantly higher numbers of enriched intronic splicing regulatory element (ISRE) motifs and other hexamer sequences (that is, potential ISRE motifs) in intron sequences flanking up exons than down exons (*P* < 10^−96^, binomial test; Fig. 4e,f), a result robust to exon downsampling (Supplementary Fig. 11 and Methods). This further supports progressive inclusion increase constituting a more regulated and functionally relevant form of devAS than inclusion decrease. However, highly conserved instances of both types exist (Fig. 2c: exon 2 is a conserved up case, exons 1 and 3 are down exons). The strong up pattern motif enrichments are found for the brain and heart, further supporting an important role of devAS in these organs. They are also found for the testis and may indicate that the transition of sexually immature to mature testis is accompanied by an at least partly regulated PSI alteration for exons in emerging spermatogenic cell types and/or somatic support cells.

Splicing factors (SFs) can have opposing effects on exon usage, depending on whether they bind to their motifs up- or downstream of the alternative exon^28^. To search for such motifs, we examined hexamer sequence frequencies for devAS events with up or down patterns. For brain and heart, we detected a significantly greater number of overrepresented hexamer sequences located upstream of up exons and downstream of down exons than expected by chance (*P* *<* 10^−10^, Fisher’s exact test; Supplementary Fig. 12). These contrasting motif localizations suggest that the corresponding SFs promote or repress exon inclusion during development, depending on the hexamer motif’s intronic position and their developmental activity dynamics, giving rise to the observed up and down developmental inclusion patterns.

We uncovered a notable case of potential opposing polarity effects of an ISRE motif between organs. A hexamer motif (ACTAAC) similar to that of the AS regulator, quaking homolog, KH domain RNA binding (QKI), which has key functions in the developing brain and heart^33,34^, is enriched upstream of up exons and downstream of down exons in the brain, whereas, in the heart, it shows the reverse pattern (Fig. 4g and Supplementary Fig. 13). QKI acts as a repressor when binding upstream and as an activator when binding downstream of a cassette exon^35^. Our observations suggest that the developmental dynamics of QKI-targeted exons not only depend on the localization of the QKI-binding motifs but also on the organ-specific developmental dynamics of their transregulatory environment, that is, the developmental expression/activity patterns of QKI and/or one or several of the SFs with which it may interact^35^ (Fig. 4g, right panel). Our results suggest that, in the brain, the activity of QKI and/or potential co-regulatory SFs decreases over time, leading to increasing inclusion frequencies of exons with upstream QKI motifs (up pattern) because of the progressively weaker repression of inclusion by QKI/co-regulators. Exons with downstream QKI motifs show the opposite behavior. By contrast, in the heart, QKI/co-regulatory activities probably increase over time, leading to decreasing inclusion frequencies of exons with upstream QKI motifs (down pattern) because of the progressively stronger repression of inclusion by QKI/co-regulators. Exons with downstream QKI motifs show the opposite pattern.

### Microexons

**Fig. 5:**
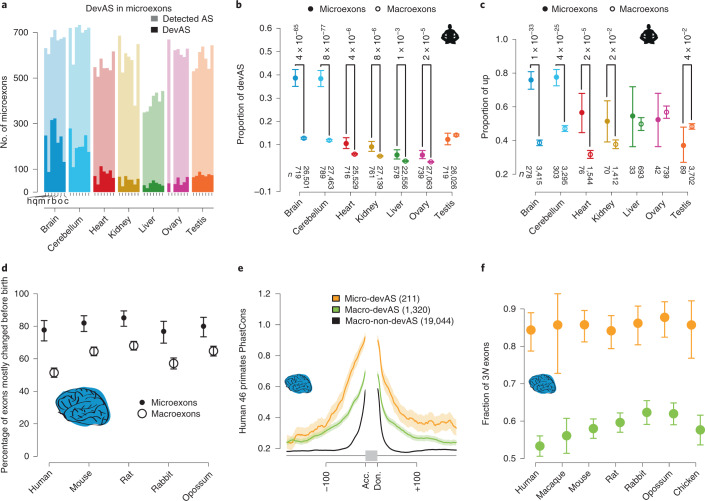
Microexons in development. **a**, Numbers of microexons (length < 28 nt) detected and with devAS across organs and species. **b**, Proportions of human micro- and macroexons with devAS among all of the cassette exons of the respective type. **c**, Proportions of exons with increasing PSI (up) among micro- and macroexons with devAS. Two-sided Fisher’s exact tests; unadjusted *P* values are shown for comparisons where *P* < 0.05. Numbers of compared segments are shown at the bottom of **b** and **c**. **d**, Proportions of up micro- and macroexons for which PSI changes occur predominantly before birth in the mouse or the corresponding stages in the other species. **e**, Mean primate sequence conservation (PhastCons^51^) scores of intronic sequences flanking human alternative micro- or macroexons with devAS or without (non-devAS). Numbers of cassette exons of the different types are indicated. Mean and 95% CIs are shown by lines and shading, respectively. Intronic donor (don.) and acceptor (acc.) sites around a schematic cassette exon (in gray) are indicated. **f**, Fractions of micro- (orange) and macroexons (green) with devAS and length divisible by three in the brain of each species. In **b**–**d** and **f**, mean values are shown by dots and error bars indicate the 95% CIs based on binomial distributions. The organ icon is from a previous study^18^.

Microexons are a special class of very short (3–27 nt) and predominantly frame-preserving cassette exons^36,37^. We found an excess of microexons with devAS in the brain compared with the other organs, thus extending observations for the adult brain^37^ (Fig. 5a,b and Supplementary Fig. 14). This excess is significantly greater than that for longer cassette exons (termed ‘macroexons’) (Fig. 5b and Supplementary Fig. 14). However, the overall excess of devAS in the brain and its specific patterns (Figs. 1c and 2a) remain very similar when restricting the analyses to the much more numerous macroexons (Supplementary Fig. 15). Albeit less pronounced, the excess of devAS events involving microexons also occurs in most other organs (Fig. 5b and Supplementary Fig. 14). The enrichment of devAS among microexons is driven by microexons with progressively increasing PSI during development (up pattern) (Fig. 5c and Supplementary Fig. 14). However, for most up microexons in the brain, the bulk of PSI increase occurs before birth (Fig. 5d). Up macroexons display significantly smaller proportions of prenatal changes in the brain than up microexons (Fig. 5d; *P* < 10^−4^ in all species, Fisher’s exact test). Overall, our observations reveal that inclusion frequencies of microexons increase during development and suggest a prominent role of microexons in early brain development. This latter notion is in agreement with microexons being neuron specific and predominantly involved in neurogenesis^37,38^, and with the misregulation of microexons being associated with autism^37^, a disorder associated with genes predominantly expressed in early brain development^39,40^.

**Extended Data Fig. 5:**
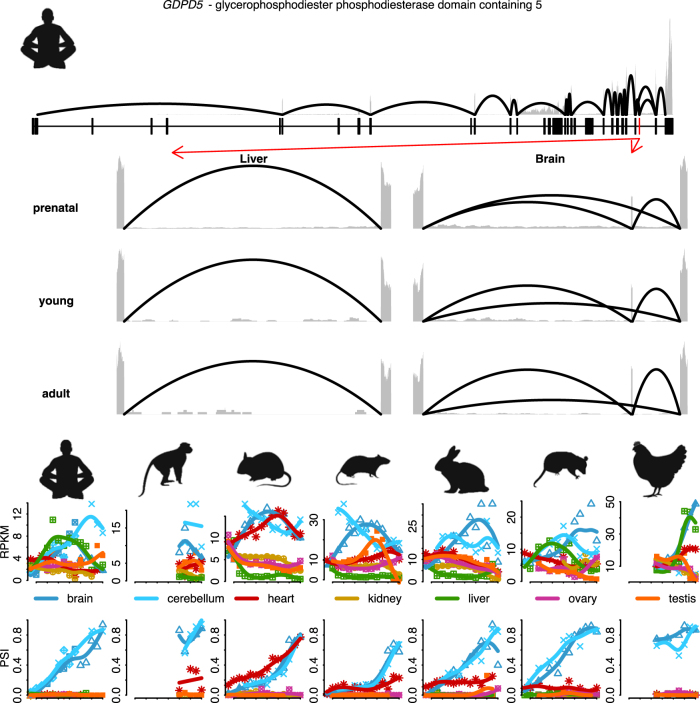
Brain-specific microexon in the *GDPD5* (Glycerophosphodiester Phosphodiesterase Domain Containing 5) gene. Total read coverage of *GDPD5* (based on human RNA-seq libraries) is shown on top. Gray lines/areas indicate the read coverage of a given genomic position; black arcs indicate reads mapped to exon-exon junctions (height of the arc is proportional to the number of mapped reads). Only junctions that fall completely within the region and with coverage greater than 5% of the coverage of the most covered junction are shown. Exons are shown by black rectangles and the alternative microexon is marked in red. Zoomed read coverages of the microexon in heart and brain for all prenatal, neonate/toddler (young), and teenager/adult (adult) samples, respectively, are shown in the middle. Gene expression levels (RPKM) and AS (PSI) profiles for individual species are shown further below. Patterns are shown for matched developmental stages^18^. Different organs are shown in different colors (color code as used throughout the paper; for example Fig. 1). The microexon is 15 nt long (coordinates: 11:75150385-75150399; genome assembly: GRCh37). Species icons (except human) are from a previous study^18^.

**Extended Data Fig. 6:**
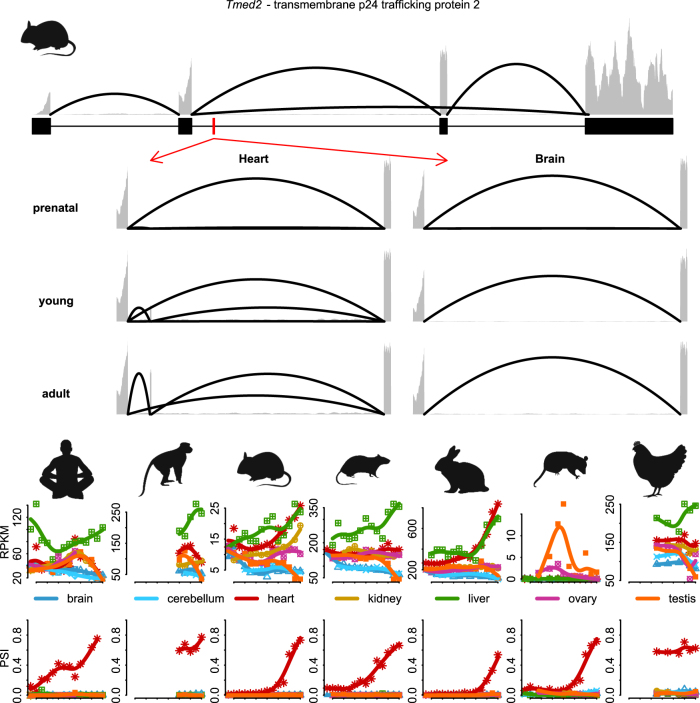
Heart-specific microexon in the *Tmed2* (transmembrane p24 trafficking protein 2) gene. Total read coverage of *Tmed2* (based on mouse RNA-seq libraries) is shown on top. Gray lines/areas indicate the read coverage of a given genomic position; black arcs indicate reads mapped to exon-exon junctions (height of the arc is proportional to the number of mapped reads). Only junctions that fall completely within the region and with coverage greater than 5% of the coverage of the most covered junction are shown. Exons are shown by black rectangles and the alternative microexon is marked in red. Zoomed read coverages of the microexon in heart and brain for all prenatal, up to 24 days postnatal (young), and from 25 days postnatal (adult) samples, respectively, are shown in the middle. Gene expression levels (RPKM) and AS (PSI) profiles for individual species are shown below. Patterns are shown for matched developmental stages^18^. Different organs are shown in different colors (color code as used throughout the paper; for example Fig. 1). The microexon is 21 nt long (coordinates: 5:124543487-124543507; genome assembly: GRCm38). Species icons (except human) are from a previous study^18^.

**Extended Data Fig. 7:**
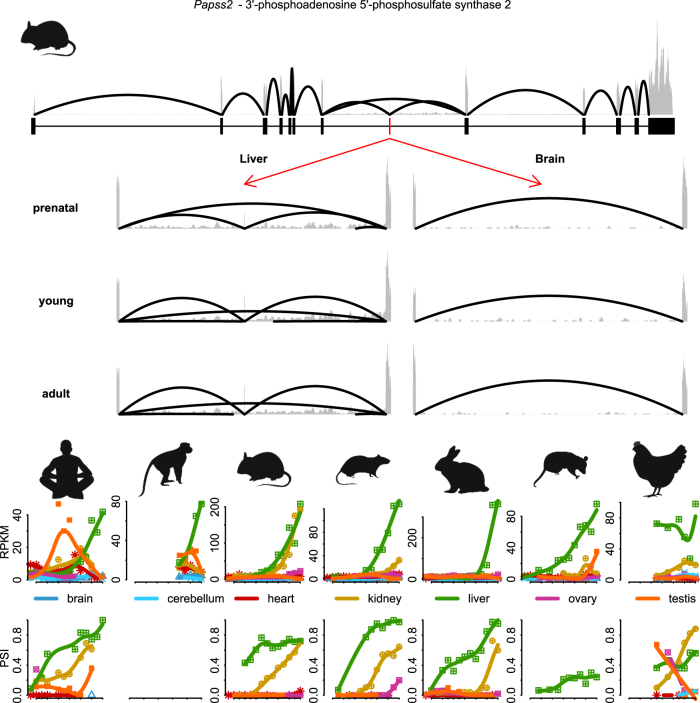
Liver-specific microexon in the *Papss2* (3’-phosphoadenosine 5’-phosphosulfate synthase 2) gene. Total read coverage of *Papss2* (based on mouse RNA-seq libraries) is shown on the top. Gray lines/areas indicate the coverage of reads mapped to a given genomic position; black arcs indicate reads mapped to exon-exon junctions (height of the arc is proportional to the number of mapped reads). Only junctions that fall completely within the region and with coverage greater than 5% of coverage relative to the most covered junction are shown. Exons are shown by black rectangles below, whereas the alternative microexon is marked in red. Zoomed read coverages of the microexon in liver and brain for all prenatal, up to 24 days postnatal (young), and from 25 days postnatal (adult) samples, respectively, are shown in the middle. Gene expression levels (RPKM) and alternative splicing (PSI) profiles for individual species are shown below. Patterns are shown for matched developmental stages^18^. Different organs are shown in different colors (color code as used throughout the paper; for example Fig. 1). The microexon is 15 nt long (coordinates: 19:32646329-32646343; genome assembly: GRCm38). Species icons (except human) are from a previous study^18^.

Previous work indicated that microexons are more conserved and functionally relevant than macroexons^37,38^. Focusing on devAS exons, we find that, indeed, microexons show higher sequence conservation in their intronic flanks than macroexons (Fig. 5e). Moreover, substantially larger proportions of microexons (~90%) with devAS preserve the reading frame when included compared with devAS macroexons (Fig. 5f). Examples of microexons with highly conserved devAS patterns in the brain, heart and liver/kidney are present in the genes *GDPD5*, *TMED2* and *PAPSS2*, respectively (Extended Data Fig. 5–7). Altogether, our findings suggest that microexons constitute a strongly selectively preserved class of alternative exons with important roles in early development, particularly that of the brain.

### New exon birth and exon alternification

**Fig. 6:**
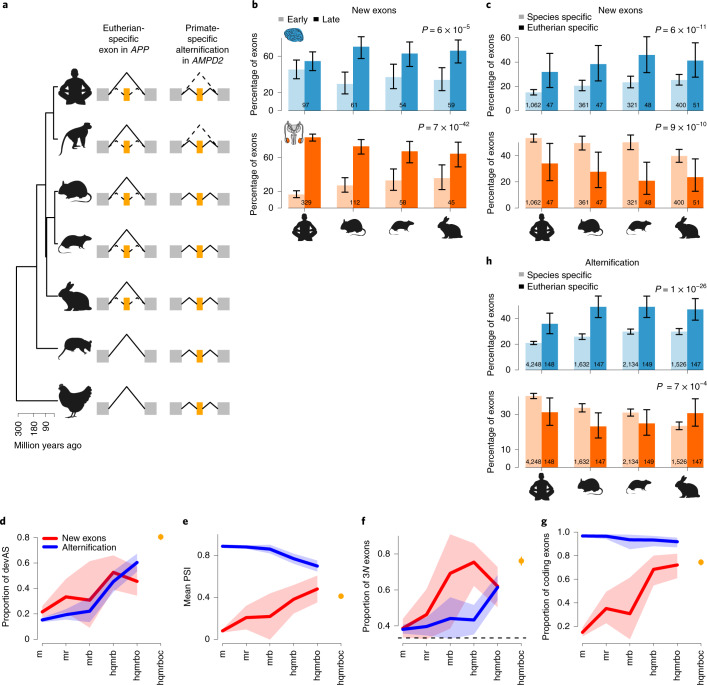
Evolution of new cassette exons. **a**, New alternative exons can originate as new cassette exons (orange, left column), or through the ‘alternification’ of ancestral constitutive exons (orange, right column). Constitutive exons are shown in gray and new splicing patterns by dashed lines. Illustrations are based on a new exon in *APP* (Extended Data Fig. 9) and an alternified exon in *AMPD2* (Extended Data Fig. 10). Left: phylogenetic tree that indicates the species carrying the new/alternified exons (that is, the evolutionary age of these events). **b**, Percentages of new eutherian cassette exons (that is, they are not present in opossum and chicken) with mean PSI values higher (light colors) or lower (dark colors) in early development (that is, prenatally in mouse and at corresponding stages in the other species^18^) than their mean PSI values in later development. Only exons with mean PSI differences > 0.1 between the two periods were considered. **c**, Percentages of new species-specific (light colors) or eutherian-specific (that is, originated in the common eutherian ancestor; darker colors) cassette exons with PSI values that are highest in the brain or testis. **d**, Proportions of new (red line) and alternified (blue line) exons with devAS that are specific to mouse (m) or are shared between mouse and different sets of species (human (h), macaque (q), rat (r), rabbit (b), opossum (o) and chicken (c)). Same analysis for ancient exons shown for comparison (orange dot). **e**, Mean PSI values of new/alternified exons. **f**, Proportions of new/alternified exons with length divisible by three. **g**, Proportions of new/alternified exons that overlap Ensembl coding sequences (Methods). **h**, Percentages of species-specific (light colors) or eutherian-specific (darker colors) alternified exons with PSI values that are lowest (that is, skipping/exclusion rates are highest) in the brain or testis. In **b**, **c** and **h**, *P* values of binomial tests are indicated (data combined across species). Error bars (**b**, **c** and **h**) and shading (**d**–**g**) indicate 95% CIs based on binomial (**b**–**d**, **f**–**h**) or normal (**e**) distributions. Exon numbers are shown inside bars in **b**, **c** and **h**. The organ and species icons (except human) are from a previous study^18^.

In adult tissues, new exons typically emerge during evolution as alternatively spliced cassette exons^5,41,42^. Thus, we assessed the origination and evolution of new (internal) cassette exons in the context of organ development (Fig. 6a, Supplementary Data 10 and Methods). We find that new alternative exons (that is, those emerging during eutherian evolution) are used more frequently late than early in development (Fig. 6b). This developmental pattern is also observed for other molecular innovations and is probably explained by progressive decreases in functional constraints during development that facilitate molecular innovations^18^. We find that very young cassette exons (that is, species specific) are predominantly incorporated into testis isoforms, as observed for adult testis^43^, whereas new exons of greater age (that is, that emerged in the ancestor of eutherians) are predominantly used in the brain (Fig. 6c). Notably, new exons of greater age also show higher proportions of devAS, mean PSI, frame preservation and coding potential (Fig. 6d–g). Overall, our findings agree with observations made for the emergence of entire new genes and the ‘out-of-testis’ scenario^42,44^. We propose that the initial transcription/splicing of new mammalian exons was facilitated by the permissive transcriptional environment of germ cells in the sexually mature testis^23,24^. Although many of these new exons remained nonfunctional and were eventually lost, a subset evolved functional roles in the testis. Over longer evolutionary periods, further mutations were fixed in these new exons, leading to their inclusion and functionality in other organs, particularly the brain.

**Extended Data Fig. 8:**
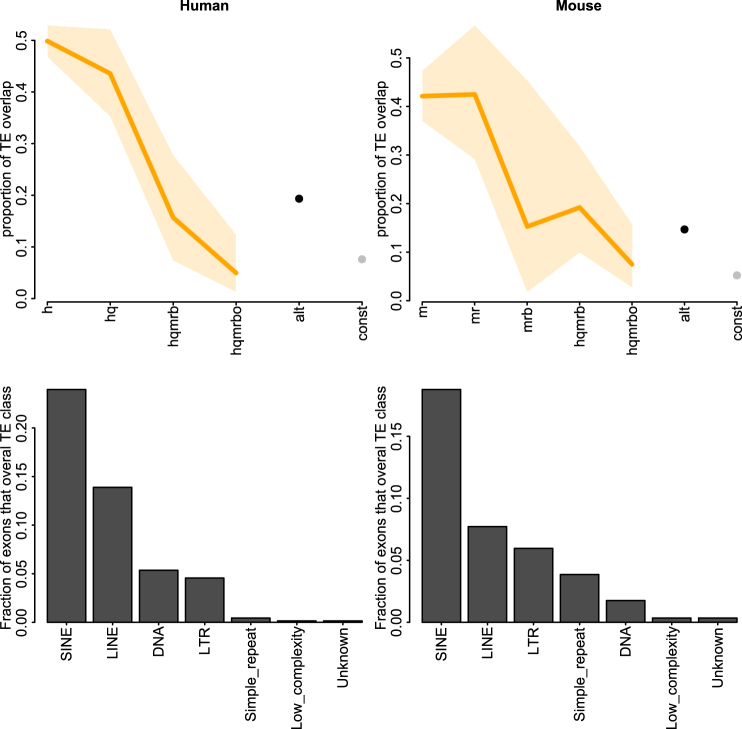
TEs in new exons. Top panels: Proportions of exons in human (left) and mouse (right) that overlap TEs for cassette exons of increasing evolutionary ages (that is, the set of species in which the exons are present). Species are denoted by letters: human (h), macaque (q), mouse (m), rat (r), rabbit (b), and opossum (o). The proportions of all alternative (alt) and all constitutive (const) exons are also shown for comparison. Lines show the mean values and shadings the 95% confidence intervals based on binomial distributions. Bottom panels: fractions of new exons that overlap TEs of a given class; TE classes are ordered by abundance and only the seven most abundant classes are shown.

**Extended Data Fig. 9:**
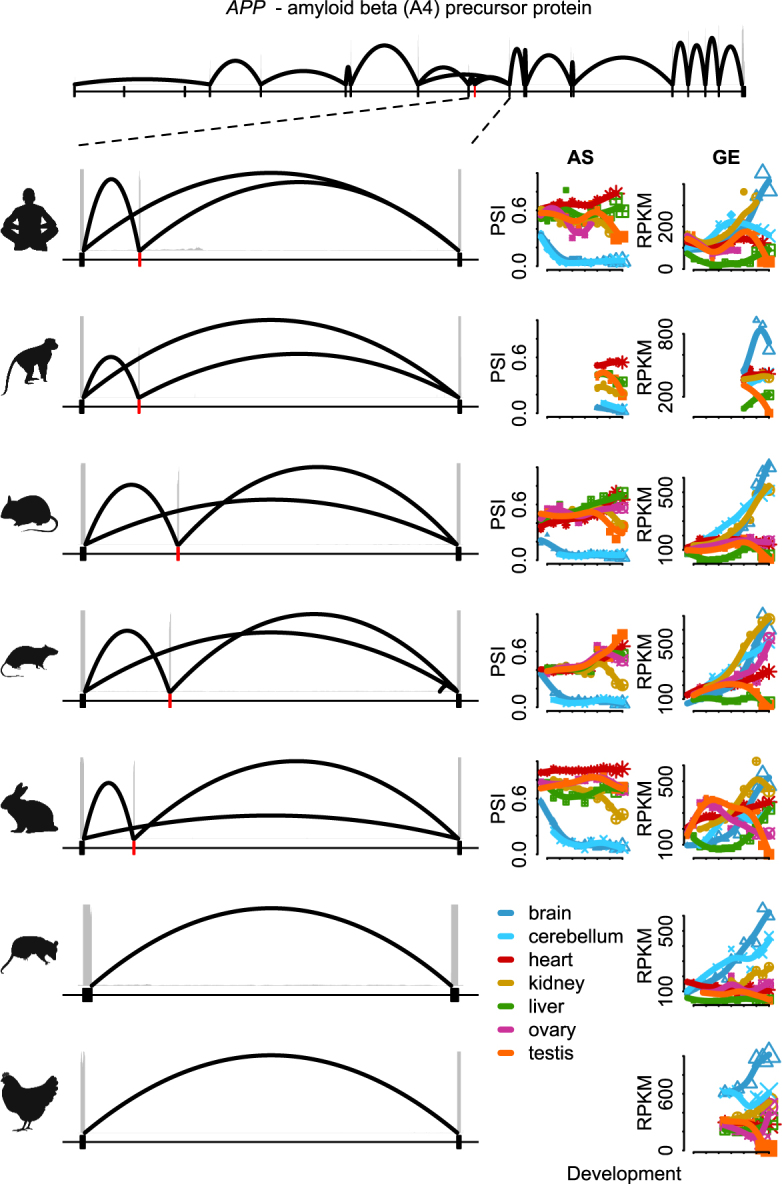
Eutherian-specific cassette exon in the *APP* (amyloid beta precursor protein) gene. Total read coverage in humans is shown on top. Gray lines/areas indicate the read coverage in a given genomic position; black arcs indicate reads mapped to exon-exon junctions (height of the arc is proportional to the number of mapped reads). Only junctions that fall completely within the region and with coverage greater than 5% of the coverage of the most covered junction are shown. Exons are shown as black rectangles, except for the eutherian-specific cassette exon, which is shown in red. Read coverage for the zoomed region is shown for all species on the left (below). Developmental patterns of AS (PSI) and gene expression (RPKM) are shown on the right. Different organs are shown in different colors (color code as used throughout the paper; for example Fig. 1). Patterns are shown for matched developmental stages^18^. The eutherian-specific exon is 57 nt long (coordinates: 21:27369675-27369731; genome assembly: GRCh37) and overlaps ‘Protein binding’ and ‘Intrinsically unstructured polypeptide region’ protein features. A nonsense mutation in this exon is associated with impaired brain development (Human Gene Mutation Database, HGMD, identifier: CM1612858; http://www.hgmd.cf.ac.uk/ac/index.php). Species icons (except human) are from a previous study^18^.

We found that ∼40–50% of species-specific new exons overlap transposable elements (TEs), particularly short interspersed nuclear elements and long interspersed nuclear elements, consistent with previous work^43,45^. We also found that the percentages of new exons overlapping TEs strongly and progressively drop with the increasing evolutionary age of exons (Extended Data Fig. 8). Our observations suggest that TE-derived exons have mostly not been selectively retained for long evolutionary periods, contrary to exons derived from unique sequences. However, it is also possible that the sequence signature of TE-derived exons changed beyond recognition over longer evolutionary periods. An example of a newly emerged alternative exon, which originated in the common ancestor of eutherian mammals, is present in the gene *APP* (Fig. 6a and Extended Data Fig. 9). A nonsense mutation in this exon, which is predominantly included in early brain development (Extended Data Fig. 9), leads to severe impairments of human brain development^46^.

**Extended Data Fig. 10:**
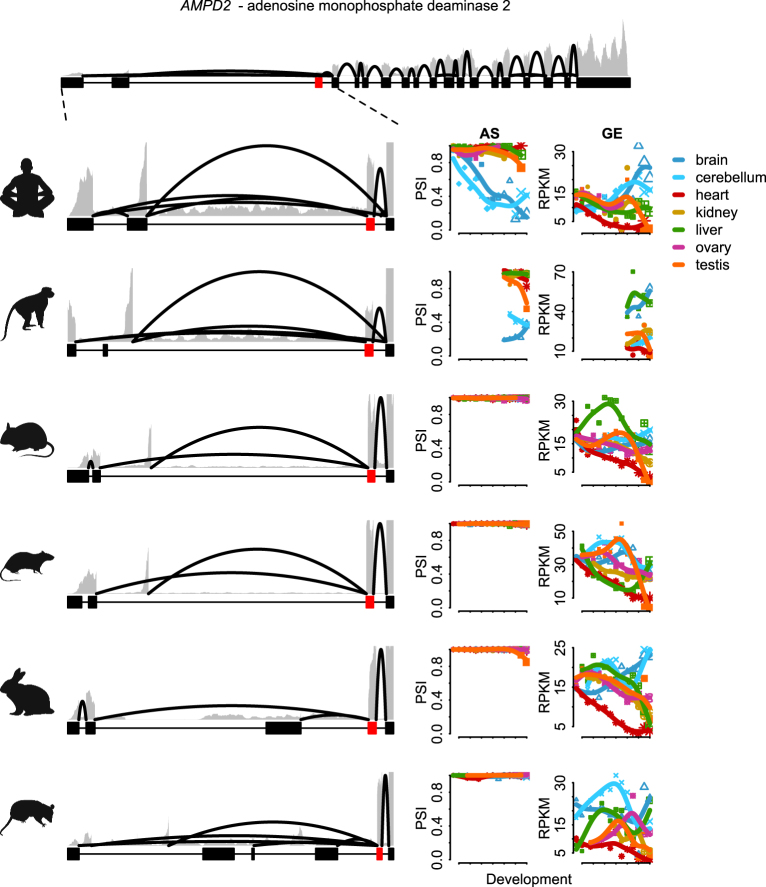
Primate- and brain-specific alternification in the *AMPD2* (adenosine monophosphate deaminase 2) gene. Total read coverage (all brain RNA-seq libraries) in humans is shown on the top. Gray lines/areas indicate the read coverage in a given genomic position; black arcs indicate reads mapped to exon-exon junctions (height of the arc is proportional to the number of mapped reads). Only junctions that fall completely within the region and with coverage greater than 5% of the coverage of the most covered junction are shown. Exons are shown as black rectangles below, except for the exon that became alternative during primate evolution, which is shown in red. Read coverage (all brain samples, respectively) for the zoomed region is shown for all species to the left (below). Developmental patterns of AS (PSI) and gene expression (RPKM) are shown on the right. Different organs are shown in different colors (color code as used throughout the paper; for example Fig. 1). Patterns are shown for matched developmental stages^18^. The alternified exon is 131 nt long (coordinates: 1:110167925-110168055; genome assembly: GRCh37) and overlaps O-phospho-L-serine, Monomethylated L-arginine, Peroxisome targeting signal, and Intrinsically unstructured polypeptide region protein features. A mutation (deletion) in this exon is associated with hereditary spastic paraplegias (HSPs); that is, neurodegenerative motor neuron diseases characterized by progressive age-dependent loss of corticospinal motor tract function (Human Gene Mutation Database^53^, HGMD, identifier: CD140996; http://www.hgmd.cf.ac.uk/ac/index.php). Species icons (except human) are from a previous study^18^.

We also investigated another evolutionary source of new alternative exons: constitutive exons. In a process that we term ‘alternification’ (Fig. 6a and Supplementary Data 11), constitutive exons evolve into cassette exons after the fixation of mutations that affect splicing^47^. Alternified exons show several parallels to new exons: a shift from testis- to brain-biased inclusion (Fig. 6h), and increased proportions of devAS (Fig. 6d) and frame-preserving exons (Fig. 6f) with increasing evolutionary age (consistent with work in adult tissues^5^). However, consistent with the fact that alternified exons stem from functional constitutive exons, and contrary to new exons, nearly all of them have coding capacity (Fig. 6g) and show a drop in mean PSI with increasing evolutionary age (Fig. 6e). These observations suggest that the more substantial and developmentally dynamic skipping of older alternification events is likely to be of functional relevance, whereas the nonfrequent exclusion of young (often frame-disrupting) exons might primarily reflect transcriptional noise. As an example, an exon in the gene *AMPD2* has become alternative only in primates (Fig. 6a and Extended Data Fig. 10). Deletions in this exon, which becomes progressively excluded during development specifically in the brain (Extended Data Fig. 10), are associated with neurodegenerative motor neuron disease^48^.

## Discussion

Our comparative analyses of developmental AS atlases across seven species revealed that devAS has been substantially more preserved during evolution than the more frequent nondynamic AS. DevAS also displays multiple features that suggest it to be overall highly enriched with functional AS events, as previously suggested^16^. However, the extent of devAS and selection patterns differ across organs, developmental periods, exon usage patterns, exon ages and types of cassette exons.

Our work provides a global view on developmental patterns of AS across vertebrate organs and species. However, it has one important limitation in that bulk-tissue RNA-seq data do not generally allow the assessment of the relative contributions of cellular composition changes versus changes in AS frequencies within cell types to the observed AS developmental trajectories—with two exceptions. First, knowing that microexons are largely neuron specific and involved in neurogenesis^37,38^ allows us to attribute our observation that microexons predominantly change in early development (Fig. 5d) to AS frequency changes in neurons. Second, the fact that the cellular composition of the testis fundamentally changes upon sexual maturation^22,23^, and our observation that this transition directly coincides with a radical shift in AS patterns (Figs. 1e and 3a, and Supplementary Figs. 4 and 6), afford a direct association of these two processes. However, such fundamental shifts in cell-type composition do not occur in the other organs. Consistently, all other organs show smooth, progressive divergences of AS programs during development, to which both changes in cell-type abundances and AS frequencies within the same cell types probably contribute (Figs. 1e and 3a, and Supplementary Figs. 4 and 6). Disentangling the precise contributions of cell composition versus cell-type intrinsic AS changes to the AS patterns observed in our study will require single-cell transcriptomic datasets that allow the reliable quantitative assessment of AS—an endeavor that is now within reach^49,50^.

## Methods

### Read mapping and annotations of transcribed regions

To annotate transcribed regions de novo across the seven studied species, we developed a pipeline (Supplementary Fig. 1), which involves four major steps, and applied it to the RNA-seq data from Cardoso-Moreira et al.^18^. In step 1, we mapped all RNA-seq reads from all libraries for each species to the corresponding genome sequences using HISAT2 (ref. ^54^) with the following parameters: --no-softclip --max-intronlen 1000000 --rna-strandness R --novel-splicesite-outfile out.ss for the first mapping and --no-softclip --max-intronlen 1000000 --rna-strandness R --novel-splicesite-infile in.ss. Genome sequences were retrieved from the Ensembl database^55^; assembly versions are listed in Supplementary Table 3.

In step 2, we extracted all intron coordinates inferred from step 1 and aligned introns between species, to maximize the number of annotated introns and resulting exon junction coordinates for downstream AS analyses. Specifically, for each species, we aligned the intronic sequences to the genomes of all other species based on pairwise whole-genome alignments. Alignments were generated using LASTZ v.1.02 (ref. ^56^) and several University of California, Santa Cruz (UCSC) tools according to genomewiki.ucsc.edu. Briefly, all genomes were split into 100-megabase portions and, then, for each species we aligned each portion to every portion in the other species using LASTZ with the following parameters: K=3000 L=3000 H=2000 Y=5000 E=55 T=2 O=600 --progress --verbosity=10 --runtime –format=axt. Alignments were transformed into chain files using the axtChain UCSC tool with the parameters: -minScore=5000 -linearGap=medium. Then we used the tools chainMergeSort, chainNet and netChainSubset to combine all chain files into a single file. Matches with the highest scores were retained. In cases where whole introns could not be aligned, we aligned their 10-nt-long ends (that is, sequences with more conserved splice sites). Cross-species mapping of sequence coordinates was performed using the htsjdk liftover library^57^. Among the introns obtained for each species, we retained those that were detected: (1) in at least four samples of a given species; (2) in at least four samples of any species and with one canonical splicing site sequence (GT–AG, GC–AG or AT–AC) in a given species; and/or (3) in at least four samples in more than one species.

In step 3, all RNA-seq reads were remapped to the new set of exon–exon junctions predicted in step 2 using HISAT2 (ref. ^54^).

In step 4, we assembled transcripts and annotated transcribed regions. Specifically, we first sorted and merged transcript alignments (BAM format; converted from the HISAT2-derived SAM files using SAMtools^57^) from the second-round mapping (step 3 above) per species–organ–stage. We note that merged alignments were used only for transcriptome assembly; the AS analysis (AS determination and quantification) was performed on individual samples. To assemble transcripts for each species–organ–stage set, we used StringTie^58^ (parameters: -f 0.1 -p 12 -j 3 -g 10 --rf) with the merged BAM files as input. Finally, all obtained GTF files for each species were merged using the StringTie merge mode with the following parameters: -p 14 -m 200 -f 0 -i. The coordinates of gene (exon/intron) structures were linked to Ensembl gene coordinates (annotation versions listed in Supplementary Table 3) using the sajrcomp command from our previously developed SAJR pipeline^15,19^.

### AS determination and quantification

AS was quantified using our SAJR pipeline^15,19^ (Supplementary Fig. 1, bottom box, and Supplementary Fig. 2). Briefly, each gene was split into segments, that is, the sequence space between two adjacent splice sites, based on the exon/intron coordinates from our annotations (see previous section; Supplementary Fig. 2). Segments were classified into constitutive segments (that is, segments that are either included (for exons) or excluded (for introns) in all transcripts of a given gene) and alternative segments (that is, segments that are included in some transcripts and excluded from others). Alternative segments were classified into different classes according to the combinations of types of splice sites that define their borders: (1) cassette exons are segments that start from acceptor sites and end with donor sites; (2) alternative acceptor (donor) segments are segments that both start and end with acceptor (donor) sites; and (3) retained introns are segments that start at a donor site and end with an acceptor site (Supplementary Fig. 2).

For each segment and each sample, we calculated the number of inclusion reads (that is, reads that overlap exons by at least 1 nt) and the number of exclusion reads (that is, reads that are mapped to exon–exon junctions that span a given segment). Reads mapped to multiple genomic locations were excluded from the analysis (that is, only uniquely mapping reads were used). PSI was calculated using the following formula:

$${\mathrm{PSI}} = \frac{{i/\left( {{\mathrm{ls}} + {\mathrm{lr}} - 1} \right)}}{{i/\left( {{\mathrm{ls}} + {\mathrm{lr}} - 1} \right) + e/\left( {\mathrm{{lr}} - 1} \right)}},$$

where *i* and *e* are numbers of inclusion and exclusion reads, respectively, and ls and lr are lengths of the segment and reads, respectively. PSI was considered as undefined if *i* + *e* < 10.

### Developmentally dynamic AS

To identify devAS, we performed statistical analyses using generalized linear models with quasi-binomial distribution (quasi-likelihood ratio test). We applied the following model for each species–organ combination:

$$\left( {i,e} \right)\approx a + a^2 + a^3,$$

where *a* is the logarithm of the number of days from conception; for postnatal samples it was calculated based on the typical gestation times of 280, 165, 20, 21, 30, 15 and 21 d for human, macaque, mouse, rat, rabbit, opossum and chicken, respectively. *P* values were adjusted using the Benjamini–Hochberg procedure^59^. All segments with at least one term with adjusted *P* value < 0.05 were considered to be significant. Tests were only performed for segments with *i* + *e* > 9 in at least 60% of samples of a given organ, and with at least four samples with PSI values that fell within the range 0.1–0.9.

### Developmental PSI and devAS pattern definition

To estimate the maximum amplitude of developmental AS change (dPSI) for a given exon in a given organ, we approximated the dependence of PSI on developmental age (logarithm of days from conception) using cubic splines with four degrees of freedom (Supplementary Fig. 9a). Splines were used to predict PSI for ages of the different samples, and dPSI was calculated as the difference between maximal and minimal predicted values (Supplementary Fig. 9a). Segments with adjusted *P* values < 0.05 and dPSI > 0.2 were considered to be devAS (Supplementary Fig. 9b). Next, PSI was interpolated into 1,000 evenly distributed age points and the difference between PSI at a given point and PSI at the previous point was calculated (PSI change). To define the direction of devAS change, four additional statistics were calculated (Supplementary Figs. 9c,d): (1) ‘up’—the sum of positive PSI changes; (2) ‘down’—the absolute value of the sum of negative PSI changes; (3) ‘up_timing’—the sum of positive PSI changes multiplied by age, then divided by the up value; and (4) ‘down_timing’—the absolute value of the sum of negative PSI changes multiplied by age, then divided by the down value. All exons with a ratio of up/(up + down) < 0.3 are classified as having a down pattern and exons with a ratio > 0.7 as up. To classify the remaining exons (that is, those with a ratio from 0.3 to 0.7), we compared up_timing and down_timing; exons with up_timing < down_timing were classified as up–down and those with up_timing > down_timing as down–up (Supplementary Fig. 9e).

To calculate numbers of exons that change PSI between adjacent developmental stages in a given organ, we considered exons that are devAS in that organ. We calculated the PSI difference for each pair of consecutive developmental stages and calculated numbers of exons with a difference > 0.2 (Fig. 3d). Numbers of genes that change at specific developmental stage were taken from Cardoso-Moreira et al.^18^.

To estimate whether most AS changes take place before or after birth (Fig. 5d), we compared PSI values across three stages: earliest, newborn (based on the mouse reference) and last (adult). Exon splicing was classified as changed before birth if the absolute dPSI value between earliest and newborn stages was higher than the absolute dPSI value between newborn and the last stage.

To assess nonrandom organ patterns of cassette exons with devAS in more than one organ (Extended Data Fig. 3), we applied two-sided Fisher’s exact tests for all exons with devAS in at least one organ for all possible organ pairs.

### MDS analysis

MDS was performed using the cmdscale function from R^60^, with the number of dimensions set to two. MDS was based on pairwise distances (1 – *r*, Pearson’s correlation coefficient) between PSI values of alternatively spliced cassette exons.

### Gene expression levels

Information about the tissue specificity of gene expression (Fig. 1f), the number of genes that change expression between two consecutive developmental stages (Fig. 3d) and the classification of genes into early and late expressed genes (Fig. 3b) was retrieved from Cardoso-Moreira et al.^18^. Statistical power to detect devAS is affected by the expression level of the gene (exon), which typically also changes during development. To exclude the influence of expression levels when analyzing devAS prevalence in early and late genes (Fig. 3b), we defined cassette exons as devAS when their dPSI > 0, without performing the additional statistical tests done for defining devAS exons in all other analyses (Developmentally dynamic AS).

### Orthologous exons

We identified 1:1 orthologous constitutive and cassette exons following our previously developed approach^16^, which is based on the chained genome alignments and akin to the procedure for introns described in the read mapping/annotation section (see above); that is, to detect orthologous exons, we mapped all annotated exons from each species to all other species. Then, we constructed lists of distinct exons (based on coordinates) for each species and again mapped these across all species, resulting in coordinates/positions of all detected exons in all species. Next, for each exon and each species, we determined the union and intersect of the exon coordinates, discarding all exons with an intersect length:union length ratio < 0.6 in at least one species. We then constructed a graph in which exons correspond to nodes, and edges were drawn between nodes if the intersect:union ratio between nodes was > 0.6 in all species. Linked components of the graph that consist of one distinct exon in each species were considered to be groups of orthologous exons. This procedure resulted in 83,888 groups of orthologous exons, of which we annotated 46,210 as alternatively spliced in at least one species (AS determination and quantification).

### Annotation of human exons with protein features

We used Exon Ontology^61^ (http://fasterdb.ens-lyon.fr/ExonOntology) to annotate human exons encoding proteins with specific features, in particular intrinsically disordered regions (IDRs; referred to as intrinsically unstructured protein regions, in the Exon Ontology database). Any feature that overlaps a given exon by at least 1 nt was assigned to the exon. To evaluate abundances of different protein features in devAS and constitutive exons (Fig. 2e), we considered only exons annotated with at least one protein feature.

### Sequence motif analyses

For all sequence motif analyses, we focused on cassette exons that are surrounded by canonical splice sites (GU–AG). For each exon, we extracted 200-nt sequences up- and downstream of introns. Then, for each of two directions of devAS (that is, up or down patterns) and sequence region (up- or downstream), we tested for the significant enrichment of 4,096 possible hexamers in exons with devAS (abs(dPSI) > 0.2), compared with the remaining ones (that is, all exons with AS except those with up/down patterns), using one-sided Fisher’s exact tests. Next, we combined the *P* values obtained for the different species using an Irwin–Hall distribution and adjusted the resulting *P* values using the Benjamini–Hochberg procedure^59^. This analysis was done to identify hexamers with evolutionarily conserved enrichment.

We compared all hexamers with known motifs from the CISBP–RNA database^52^. For each motif with a position weight matrix from CISBP–RNA and each hexamer, we calculated the probability of the hexamer being generated by the motif as:

$$p\left( {{\mathrm{hex}}|{\mathrm{motif}}} \right) = 1 - \mathop {\prod }\limits_{o = 1 - {\mathrm{lm}}}^5 \left( {1 - p_o} \right),$$

where *p*_*o*_ is the probability of the motif to generate the hexamer with offset *o* and lm is the motif length. We calculated the probability of the motif to generate the hexamer with a given offset as:

$$p_o = \mathop {\prod }\limits_j f_{j - o}\left( {nt_j} \right) \times 0.25^m,$$

where *nt*_*j*_ is a *j*th nucleotide of the hexamer, *f*_*j-o*_*(nt)* is the fraction of the nucleotide in the motif position j-o, *j* runs through all hexamer positions that overlap motif with a given offset and *m* is the number of hexamer positions that do not overlap with the motif. For each motif the hexamer with highest probability was identified. All hexamers that have probability not lower than half of maximal probability were annotated with the given motif.

### Human SNP analyses

To evaluate selective constraints of potential regulatory elements near exons with devAS in humans (Fig. 4d), we used the Genome Aggregation Database^62^ (gnomAD). We collected all nonsingleton SNPs 50 nt up- and downstream of introns and compared minor allele frequencies (MAFs) between exons with up/down patterns using Mann–Whitney *U*-tests. In accordance with population genetic principles, we assumed that lower MAFs are associated with higher selective constraints^62^.

### Exon gain/loss analyses

Exons present only in subsets of species were considered as candidates for evolutionary exon ‘birth’ or loss. To identify such cases, we searched for pairs of consecutive 1:1 orthologous exons (border exons) that, in a subset of species, are interspaced by an additional exon. These additional exons formed an initial list of potentially gained or lost exons. If, for a given pair of border exons, an additional exon was observed in more than one species, we assessed the sequence similarity of these exons between species. To do so, we used BLASTN v.2.9.0+ (ref. ^63^; parameters: -word_size 8 -evalue 10000) to align exons from species where they were found to the corresponding regions in all other species (that is, the sequence of the exon plus four flanking nucleotides were used as query sequences). As potential target sequences in the other species, we used the two orthologous border exons and the sequences between them. The following filters were applied to the initial list of potentially gained/lost exons: (1) potentially gained/lost exons should not align to consecutive orthologous border exons in species where no interspaced exons were detected; (2) potentially gained/lost exons observed in multiple species should all align to each other; and (3) only exons shorter than 500 nt were considered. The type of event (gain or loss) and its evolutionary age were identified based on parsimony (that is, the scenario requiring the smallest number of evolutionary events is assumed to be the correct one); only cases that could be explained by a single gain or loss event were retained for downstream analyses. The list of new exons is provided in Supplementary Data 10. For the assessment of overlaps of human/mouse exons with TEs, a minimum overlap of 10 nt was required. TE information and coordinates were retrieved from the UCSC Genome Browser (https://genome.ucsc.edu).

### Alternification

To identify cases of evolutionary gain or loss of exon skipping. that is, the transformation from constitutive to cassette exon (‘alternification’) or reverse. we considered all 46,210 exons with orthologues across all seven species that are alternative in at least one species. We considered an exon as a cassette exon in a given species if there were at least four organ–stage pairs in which the exon had PSI values < 0.9, unless the exon was considered to be constitutive. Akin to the exon gain/loss analysis, the type of event (gain or loss) and its evolutionary age were identified based on parsimony (only single-event cases were considered). The list of new ‘alternified’ exons is provided in Supplementary Table 11.

### Data analysis

All statistical analyses and plots were done in R (v.3.3.1) as implemented in Rstudio (v.1.0.136). Plots were created using the R basic graphics. The following R packages were used: GenomicAlignments^53^ (v.1.24), reshape^64^ (v.0.8.8), png^65^ (v.0.1–7), ape^66^ (v.5.3) and seqinr^67^ (v.3.6–1).

### Reporting Summary

Further information on research design is available in the Nature Research Reporting Summary linked to this article.

## Online content

Any methods, additional references, Nature Research reporting summaries, extended data, supplementary information, acknowledgements, peer review information; details of author contributions and competing interests; and statements of data and code availability are available at 10.1038/s41588-021-00851-w.

## Supplementary information

Supplementary Information Supplementary Figs. 1–15

Reporting Summary

Peer Review Information

Supplementary Data 1–11 Supplementary Data 1. De novo transcribed-region annotations for mice in GTF format. Supplementary Data 2. De novo transcribed-region annotations for humans in GTF format. Supplementary Data 3. De novo transcribed-region annotations for macaque in GTF format. Supplementary Data 4. De novo transcribed-region annotations for rats in GTF format. Supplementary Data 5. De novo transcribed-region annotations for seven rabbits in GTF format. Supplementary Data 6. De novo transcribed-region annotations for opossums in GTF format. Supplementary Data 7. De novo transcribed-region annotations for chickens in GTF format. Supplementary Data 8. List of 1:1 orthologous segments across species and their genomic coordinates. Supplementary Data 9. Table contains all segments from all species that passed thresholds in at least one organ. The first seven columns give the segment unique ID, Ensembl ID of the genes that host the segment (if available), genome coordinates of the segment and segment type. CE. cassette exon; AA, alternative acceptor site; AD, alternative donor site; RI, retained intron. The next 21 columns provide the developmental patterns. ‘-’, segment did not pass the thresholds in a given organ; ‘n’, segment is not devAS; ‘u’, ‘d’, ‘ud’, ‘du’. denote up, down, up–down and down–up patterns, respectively. The dPSI and Benjamini–Hochberg (BH)-adjusted *P* values (segments that didn’t pass thresholds are marked by NA) for all seven organs. Supplementary Data 10. List of new cassette exons. For each new cassette exon three exon IDs are provided: seg.id is the id of the new exon; it is not applicable (NA) if the exon is not found in a given species; useg.id and dseg.id are the IDs for the upstream and downstream exons, respectively. Orth.id groups exons into their orthology group. Information about mutations within the new exon or within the 200-nt region around the new exon from the Human Gene Mutation Database^51^ (HGMD) is provided when available. Supplementary Data 11. List of new ‘alternified’ exons. List of orthologous exons that are newly alternatively spliced in at least one species. The species in which exons are alternatively spliced are listed in the column ‘alternative.in’: human, macaque, mouse, rat, rabbit, opossum, chicken. Information about mutations within the new exon or within the 200nt region around the new exon from the HGMD^51^ is provided when available.

Supplementary Tables 1–3 Supplementary Table 1. Overlap of the de novo annotations with Ensembl annotations for all AS classes. Supplementary Table 2. Numbers of genes that show significant changes in devAS during the main periods of developmental change, gene expression or both. Supplementary Table 3. Genome assembly and Ensembl annotation versions.

## Data Availability

All data are available in the main text, supplementary materials and/or accompanying database (https://apps.kaessmannlab.org/alternative-splicing). We used data from Ensembl (https://www.ensembl.org/index.html), Human Gene Mutation Database (http://www.hgmd.cf.ac.uk/ac/index.php), Exon Ontology database (http://fasterdb.ens-lyon.fr/ExonOntology), CISBP–RNA (http://cisbp-rna.ccbr.utoronto.ca/index.php) and gnomAD (https://gnomad.broadinstitute.org).

## Code availability

All custom code used in this study is available at https://github.com/iaaaka/evo-devo.
