# Supplementary material for: Alternative splicing during mammalian organ development
Source: Nat Genet. 2021 May 3;53(6):925–34. doi: 10.1038/s41588-021-00851-w (PMC8187152; doi:10.1038/s41588-021-00851-w)
Supplement: Supplementary file 1 — Supplementary Figs. 1–15 [file 41588_2021_851_MOESM1_ESM.pdf]

nature portfolio
https://doi.org/10.1038/s41588-021-00851-w

Supplementary information

Alternative splicing during mammalian organ development

In the format provided by the authors and unedited

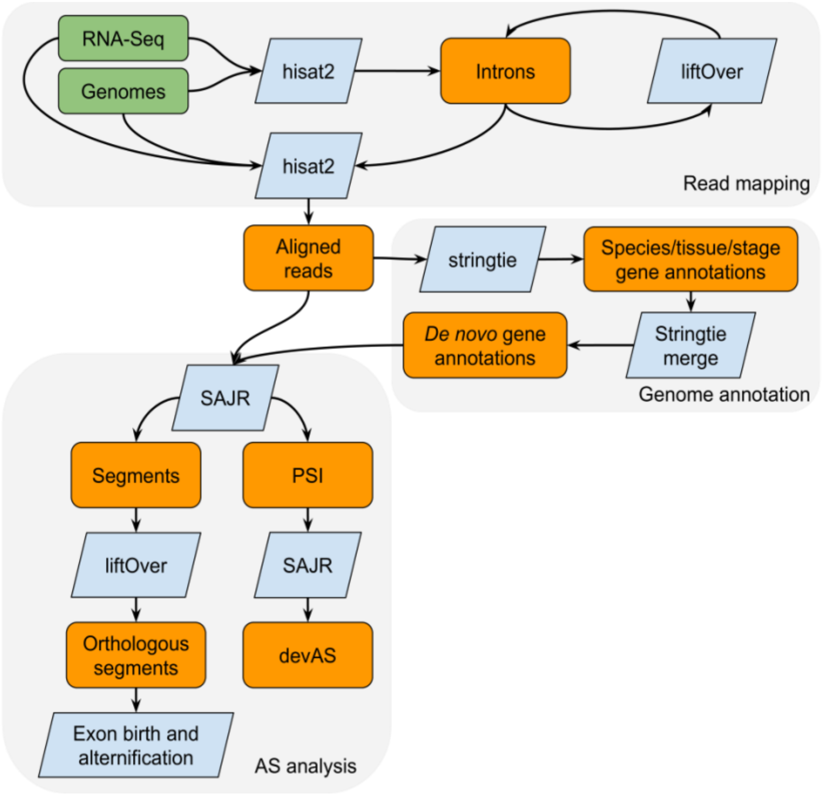

Supplementary Fig. 1 | Overview of the pipeline used to annotate gene structures and AS across species. The upper two boxes illustrate procedures for read mapping, transcriptome assembly, and annotation of gene structures/transcribed regions. Details are described in the Methods (section: Read mapping and annotations of transcribed regions). The bottom box summarizes the procedure for the determination and quantification of AS across species, which is illustrated in more detail in Supplementary Fig. 2. Details of the procedure are explained in the Methods (section: AS determination and quantification).

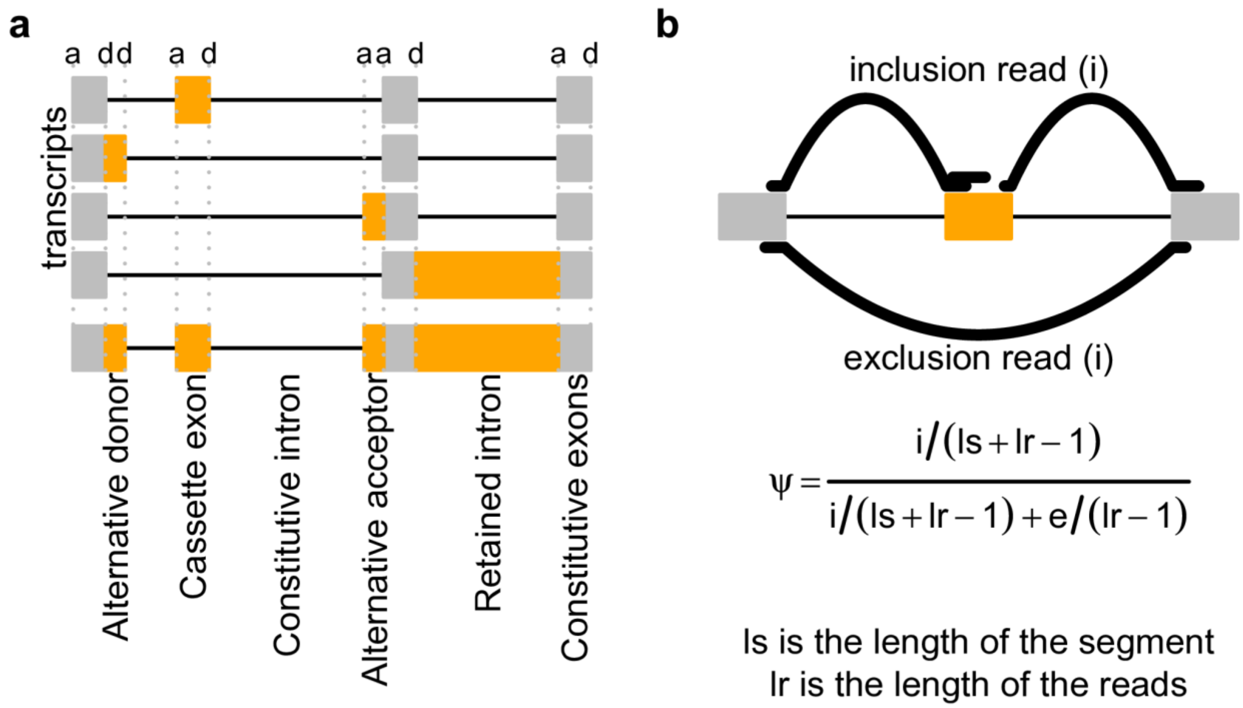

Supplementary Fig. 2 | Overview of the procedure underlying determination and quantification of AS. a, Segment definition. Different transcripts of the same gene are shown, splice sites are indicated by vertical dashed lines, acceptor (a) and donor splicing sites (d) are marked. Segments are indicated and labeled below. If a segment is always included or excluded from the transcript isoform, it is marked as a constitutive exon (gray box) or intron, respectively. Alternative segments (orange) are defined as those included in some but not all transcripts. There are four major types of segments: cassette exons (start with acceptor and end with donor sites), alternative donor or acceptor segments (defined by two alternative donor or acceptor sites, respectively), and retained introns (start with donor and end with acceptor sites). **b**, AS quantification. Two types of reads are counted for each segment: inclusion reads (overlap the segment by at least one nucleotide) and exclusion reads (map to the exon-exon junction that spans the exon). Reads that overlap constitutive or retained introns are not considered. Normalized inclusion and exclusion read counts are used to calculate percent spliced in (PSI) values. Details of the procedure are described in Methods (section: AS determination and quantification).

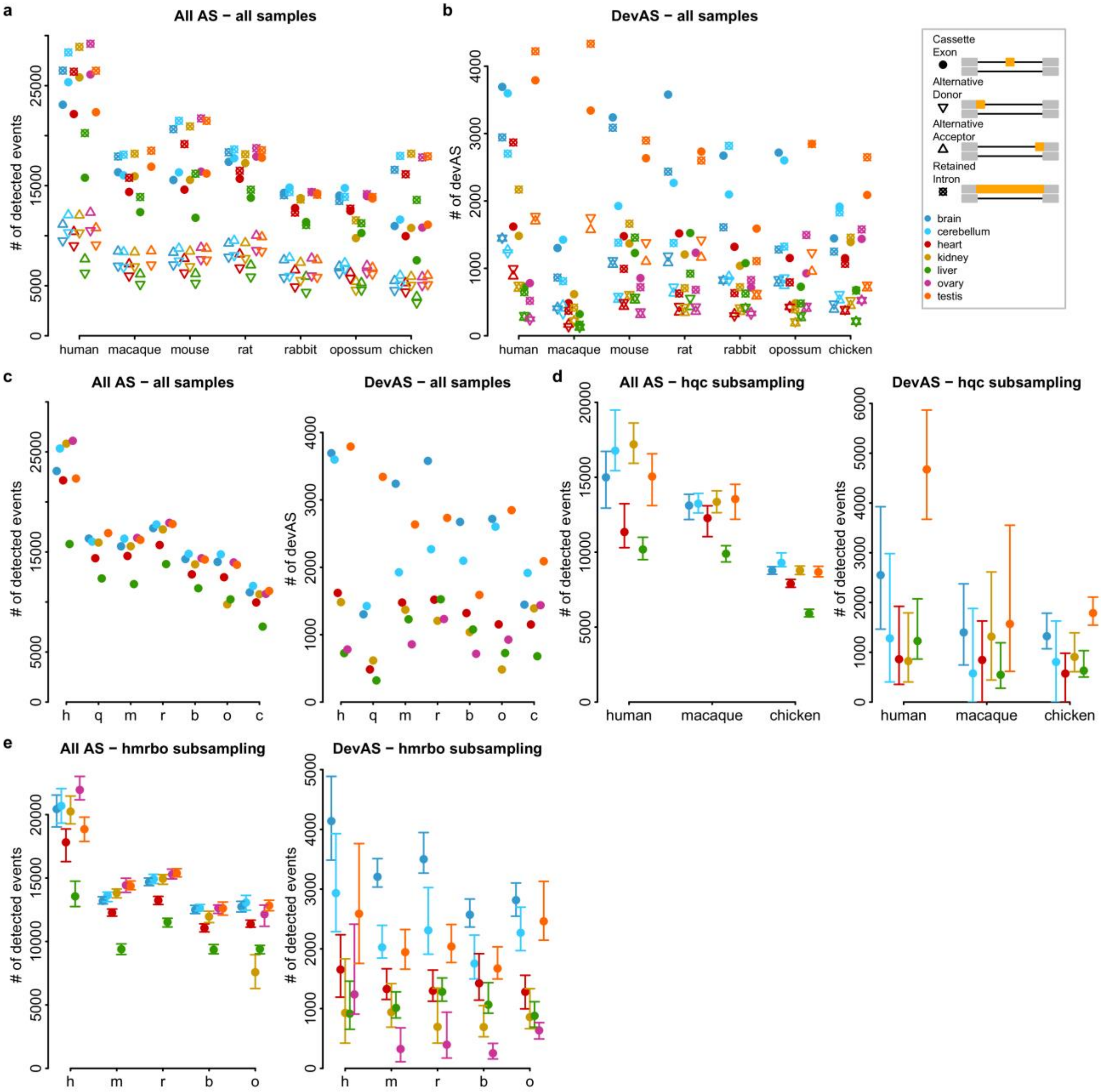

Supplementary Fig. 3 | Developmental AS atlases. a, Numbers of detected AS events for the four major classes across organs and species. Differs from Fig. 1a by showing the data grouped by species rather than by organ. **b**, Numbers of devAS events for the four major classes across organs and species. Differs from Fig. 1b by showing the number of events instead of percentages and by showing the data grouped by species rather than by organ. **c**, Numbers of detected AS (left) and devAS (right) cassette exons across organs and species (same order as in **(a)**). This is a subset of the data in **(a)** and **(b)** and is shown to facilitate comparisons with **(d)** and **(e)**. **d,** Numbers of detected AS (left) and devAS (right) cassette exons identified based on subsampling analyses for human, macaque, and chicken. **e**, Numbers of detected AS (left) and devAS (right) cassette exons identified based on subsampling analyses for human (h), mouse (m), rat (r), rabbit (b), and opossum (o). In **(d)** and **(e)**, subsampling was done to ensure the numbers of developmental stages and replicates were precisely matched across the organs and groups of species analyzed (based on the stage alignments shown in Extended Data Fig. 1). Dots and error bars in **(d)**-**(h)** show medians and the 95% confidence intervals obtained by bootstrapping (number of samples: N=100), respectively.

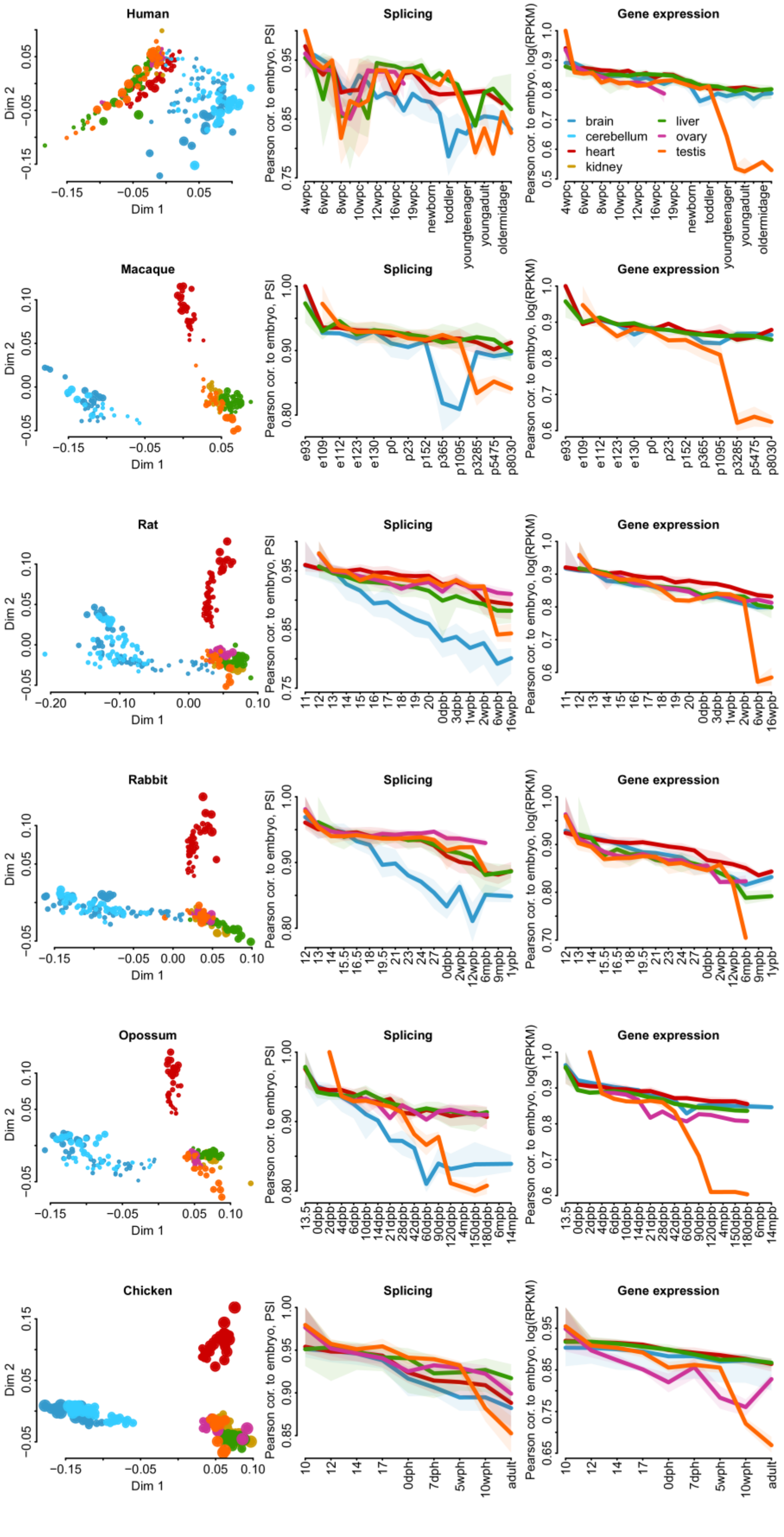
Human
Macaque
Rat
Rabbit
Opossum
Chicken
Splicing
Gene expression
Dim 1
Dim 2
Pearson cor. to embryo, PSI
Pearson cor. to embryo, log(RPKM)
brain
cerebellum
heart
kidney
liver
ovary
testis
4wpc
6wpc
8wpc
10wpc
12wpc
16wpc
19wpc
newborn
toddler
youngteenager
youngadult
oldermidage
e93
e109
e112
e123
e130
p0
p23
p152
p365
p1095
p3285
p5475
p8030
0dpb
3dpb
1wpb
2wpb
6wpb
16wpb
12wpb
6mpb
9mpb
1ypb
4mpb
14mpb
0dph
7dph
5wph
10wph
adult

Supplementary Fig. 4 | Developmental AS dynamics. Left column: MDS plots for all samples in each species based on pairwise distances (1 – r, Pearson correlation coefficient) between PSI values of all alternatively spliced cassette exons. Middle and right columns: Pearson correlation coefficient (r) of cassette exon inclusion frequencies (PSIs) (middle) and gene expression levels (right), between the earliest and all successive developmental stages for different organs in each species. Median is shown by lines, and shadings indicate the 95% confidence intervals based on bootstrapping analysis (1,000 replicates, with all possible pairs of samples between given and earliest stage considered). Different organs are shown in different colors (color code as used throughout the paper; e.g. main Fig. 1). This figure complements main Fig. 1d, e.

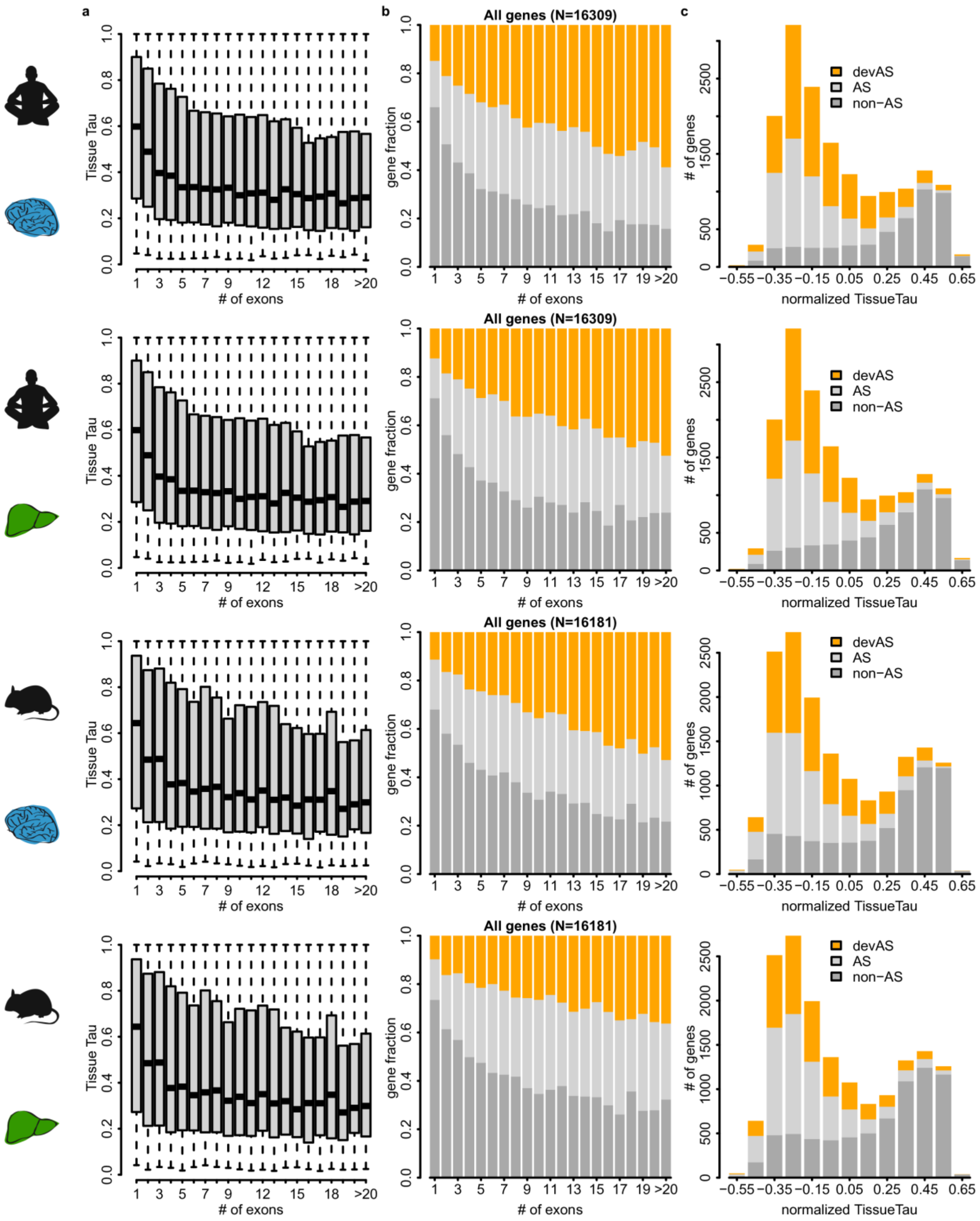

Supplementary Fig. 5 | Interplay between gene expression breadth, number of internal exons, and devAS. a, Distribution of tissue-specificity (Tissue Tau) for genes with an increasing number of internal exons. The last box includes genes with more than 20 exons. Box plots depict the median ± 25th and 75th percentiles, with whiskers at 1.5 times the interquartile range. **b**, Proportions of genes with devAS (dPSI > 0.2), non-dynamic AS (AS), or no alternatively spliced cassette exons (non-AS) for genes with an increasing number of internal exons. The last bar includes genes with more than 20 exons. **c**, Numbers of genes with devAS, AS, and non-AS cassette exons for different bins of Tissue Tau normalized by the number of internal exons. The Tissue Tau for each gene was normalized by subtracting the mean

Tissue Tau for genes with the same number of internal exons. Data for the human and mouse brain and liver are shown in different rows. Organs an species icons (except human) are from a previous study[18].

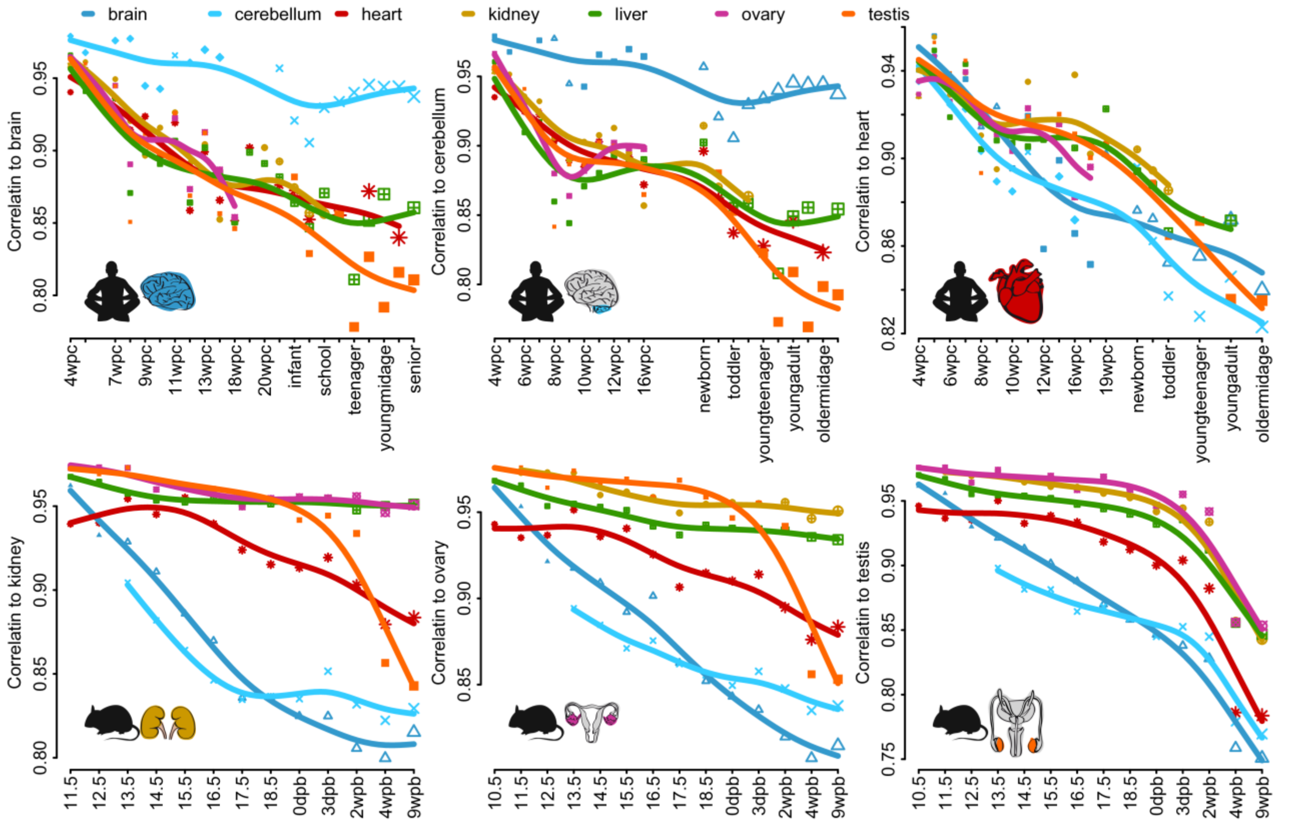

Supplementary Fig. 6 | Divergence of AS programs between organs during development. Pearson correlation coefficients for comparisons of PSI values of cassette exons between different human/mouse organs and the remaining organs across developmental stages. This figure complements main Fig. 3a. Organ and species icons (except human) are from a previous study[18].

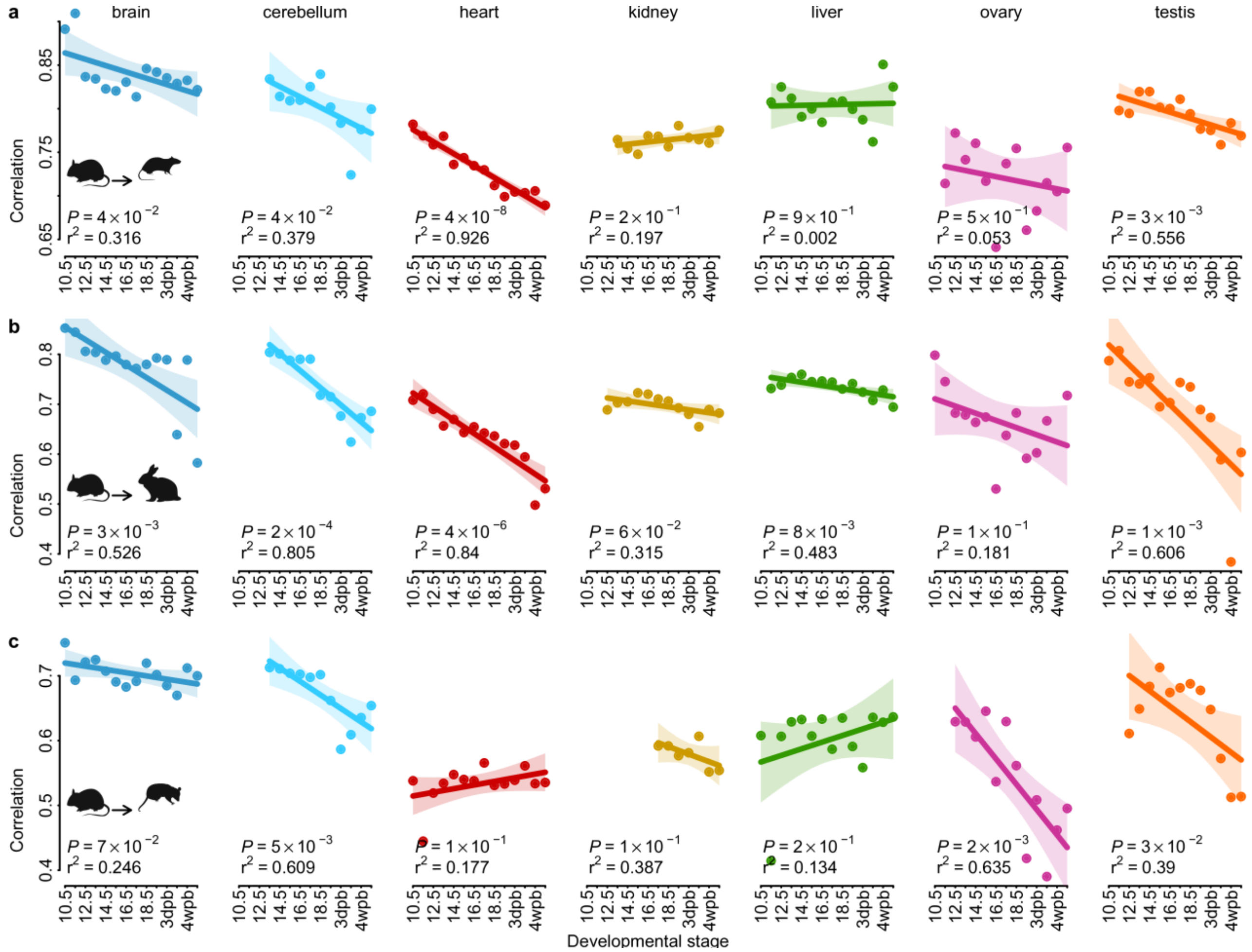

Supplementary Fig. 7 | Between-species divergence of AS across development. Pearson correlation coefficients for comparisons of PSI values of orthologous cassette exons between different species across development in the different organs (only exons with AS in both species that are devAS in at least one of them are considered: **(a)** mouse-rat, **(b)** mouse-rabbit and **(c)** mouse-opossum. Linear fit and 95% confidence intervals are shown by line and shading, respectively. Unadjusted ANOVA *P*-values and r^2 for fits are shown on each panel. This figure complements main Fig. 3c. Species icons (except human) are from a previous study[18].

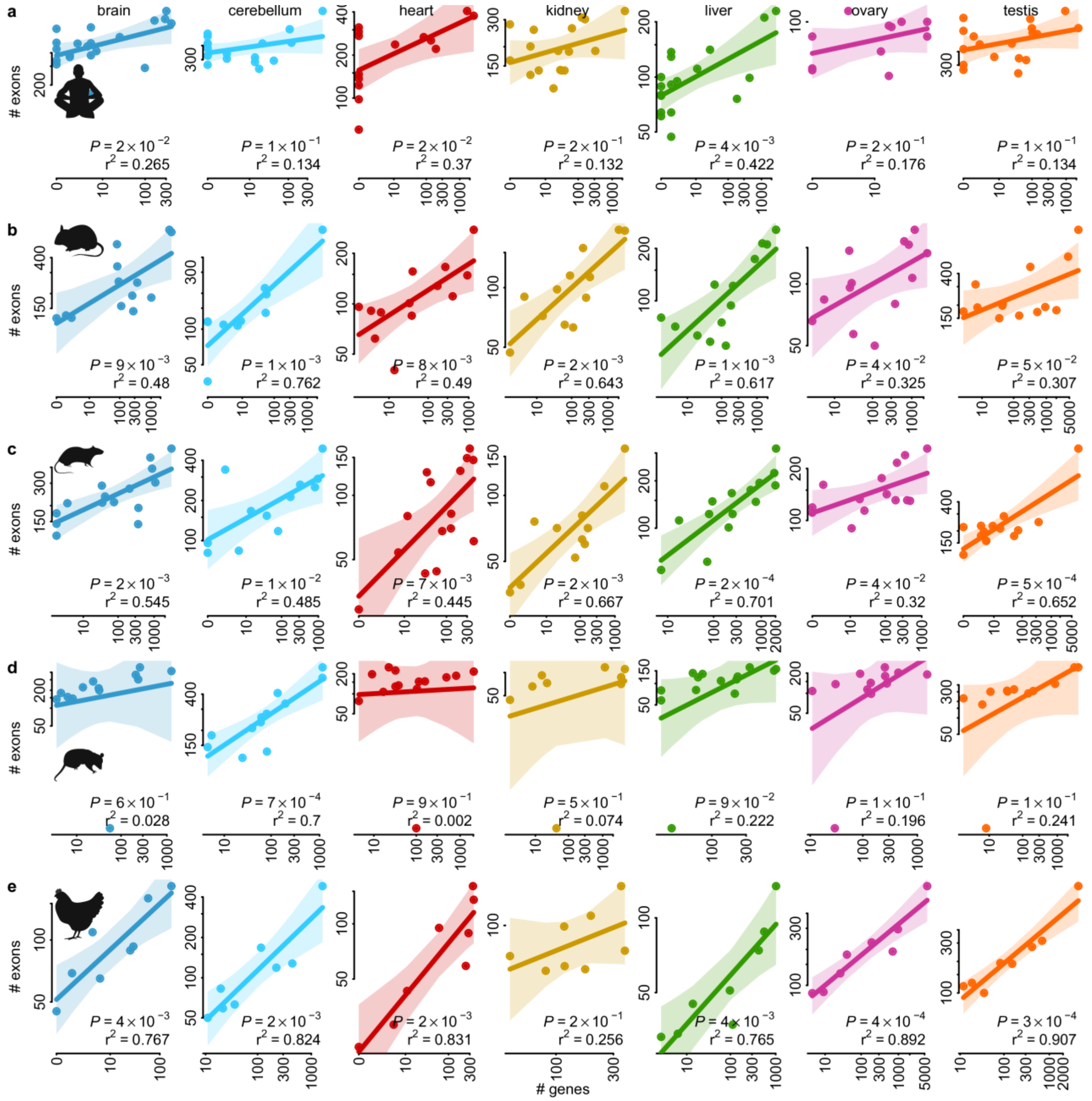

Supplementary Fig. 8 | Peaks of developmental change for devAS and gene expression levels. Correlation between numbers of genes (x-axis) and cassette exons with devAS (y-axis) that changed in expression and PSI (dPSI > 0.2), respectively, between consecutive developmental stages in human (**a**), mouse (**b**), rat (**c**), opossum (**d**), and chicken (**e**). Linear fit and 95% confidence intervals are shown by line and shading, respectively. Unadjusted ANOVA *P*-values and r^2 for fits are shown on each panel. This figure complements main Fig. 3d. Species icons (except human) are from a previous study[18].

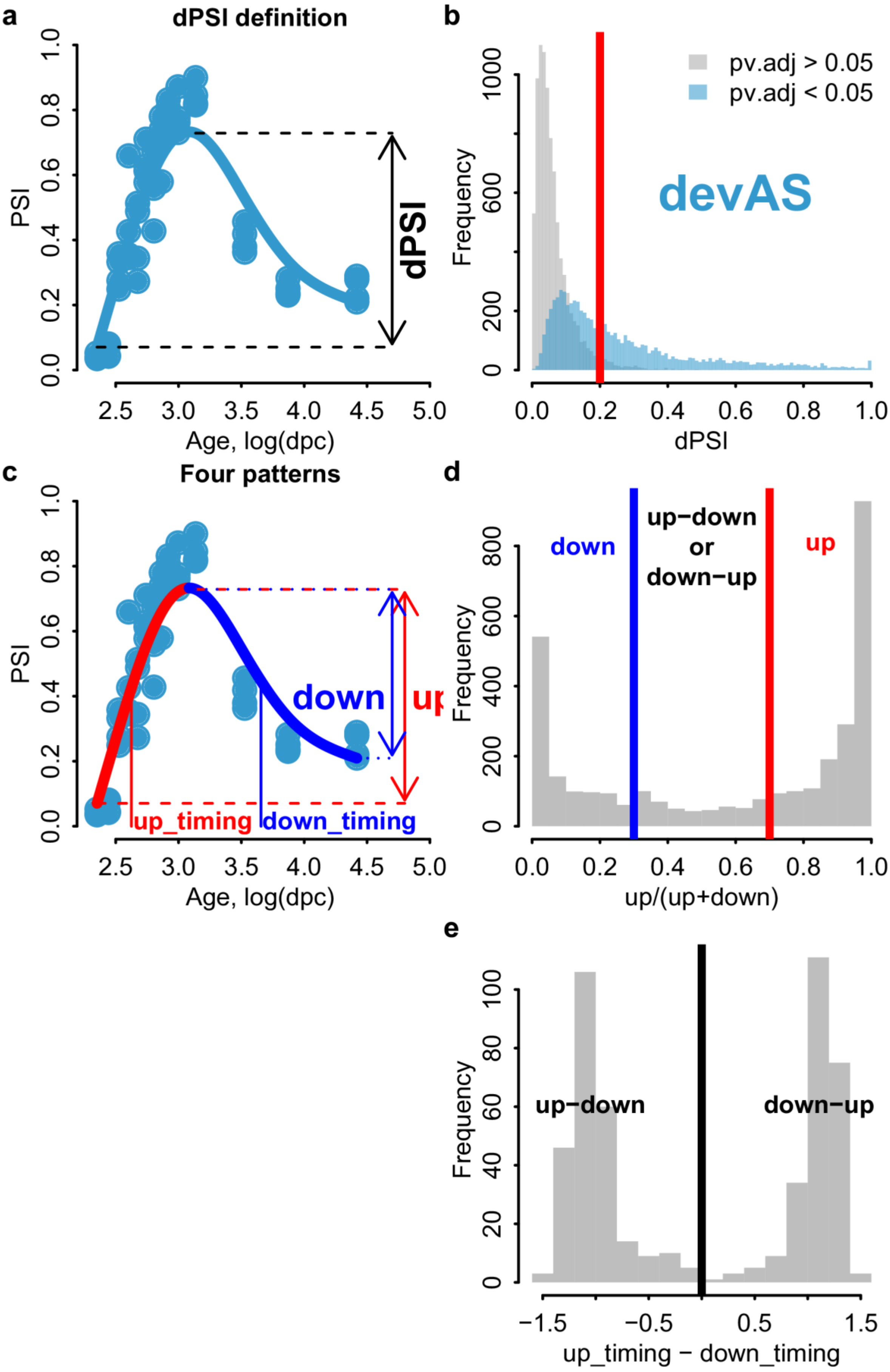
a
dPSI definition
PSI
dPSI
Age, log(dpc)
b
pv.adj > 0.05
pv.adj < 0.05
devAS
Frequency
dPSI
c
Four patterns
PSI
down
up_timing
down_timing
Age, log(dpc)
d
down
up−down
or
down−up
up
Frequency
up/(up+down)
e
up−down
down−up
Frequency
up_timing − down_timing

Supplementary Fig. 9 | dPSI and devAS pattern definition. **a**, Approximation of the dependence of PSI on developmental age (logarithm of days from conception) using cubic splines with four degrees of freedom. Splines were used to predict PSI for ages of the different samples, and dPSI was calculated as the difference between maximal and minimal predicted values. **b**, Segments with Benjamini-Hochberg adjusted *P*-values below 0.05 and dPSI > 0.2 were considered as devAS. PSI was interpolated into 1,000 evenly distributed age points and the difference between PSI at a given point and PSI at the previous one was calculated (PSI change). **c**, To define the direction of devAS change, four additional statistics were calculated: 1) "up" – the sum of positive PSI changes; 2) "down" – the absolute value of the sum of negative PSI changes; 3) "up_timing" – the sum of positive PSI changes multiplied by age, then divided by the "up" value; and 4) "down_timing" – the absolute value of the sum of negative PSI changes multiplied by age, then divided by the "down" value. **d**, Distribution of the ratio of up to sum of up and down is shown on panel. All exons with the ratio of up/(up+down) below 0.3 are classified as having a "down" pattern, exons with the ratio above 0.7 are classified as "up". **e**, To classify the remaining exons (i.e., those with a ratio from 0.3 to 0.7), we compared up_timing and down_timing; exons with up_timing < down_timing were classified as up-down, those with up_timing > down_timing as down-up. See also Methods (section: dPSI and devAS pattern definition).

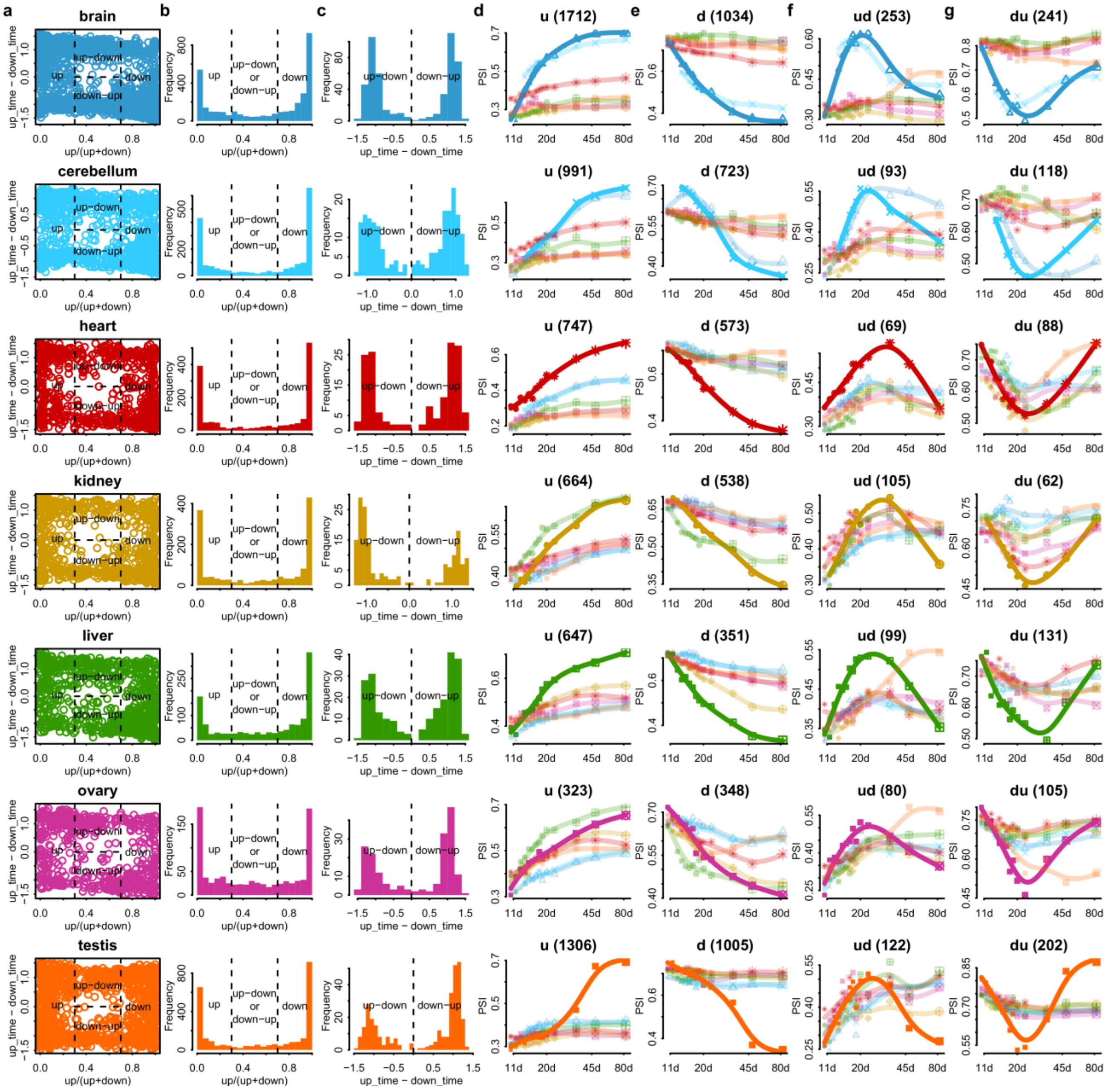

Supplementary Fig. 10 | Developmental exon usage patterns in the mouse. Illustration of the classification of mouse cassette devAS exons into four patterns. a, Dependence of up and down timing differences on up/(up+down) (see Supplementary Fig. 9 and Methods' section "dPSI and devAS pattern definition"). One data point corresponds to one exon. Thresholds are indicated by dashed lines and the patterns defined are labeled accordingly. **b**, Distribution of devAS cassette exons based on up/(up+down) ratio. **c**, Distribution of devAS cassette exons with up/(up+down) values between 0.3 and 0.7 based on timing difference. **d-g,** average PSI patterns for the four defined devAS classes in the different mouse organs. The numbers of cassette exons in each class are indicated (Methods). This figure complements main Fig. 4a.

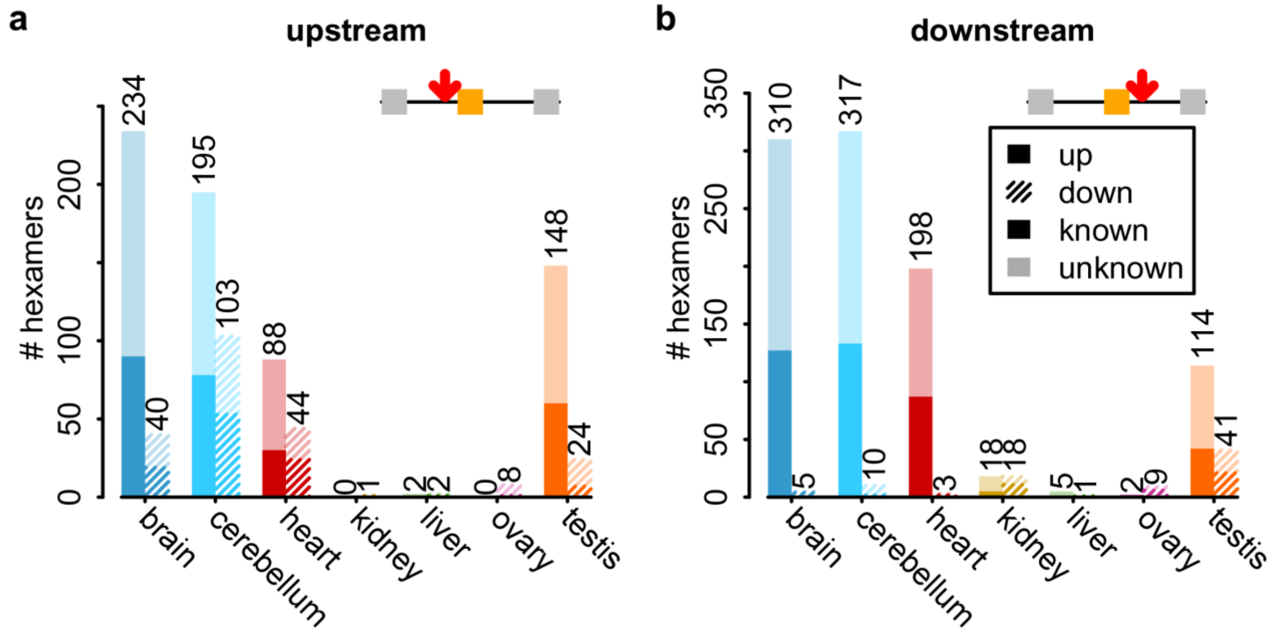

Supplementary Fig. 11 | Hexamer downsampling analysis. a-b, Numbers of hexamers significantly enriched in upstream **(a)** or downstream **(b)** intronic sequences flanking devAS exons. "Up" and "down" exons are shown by filled and hatched bars, respectively. Hexamers closely resembling known binding motifs of RNA-binding proteins from the CISBP-RNA database[37] are shown by brighter colors. This figure is similar to main Fig. 4e, f, but in this case before the hexamer enrichment analysis, the exons were downsampled to ensure that the number of "up" exons equals that of "down" exons in each species and organ.

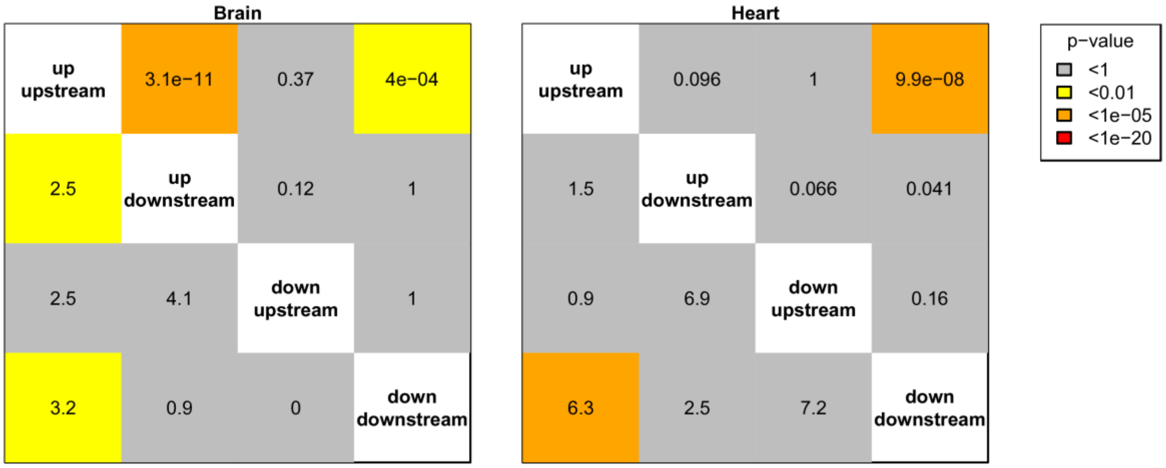

Supplementary Fig. 12 | Positional effects of binding of SFs. Overlap between hexamers significantly overrepresented in intronic sequences upstream or downstream of cassette exons with "up" or "down" patterns in the brain (left) and heart (right). Upper-right triangles show *P*-values (Two-sided Fisher's exact tests) for overlap between two sets of hexamers, while lower-left triangles show odds ratios. Only hexamers that were significant in at least one comparison in at least one organ were considered.

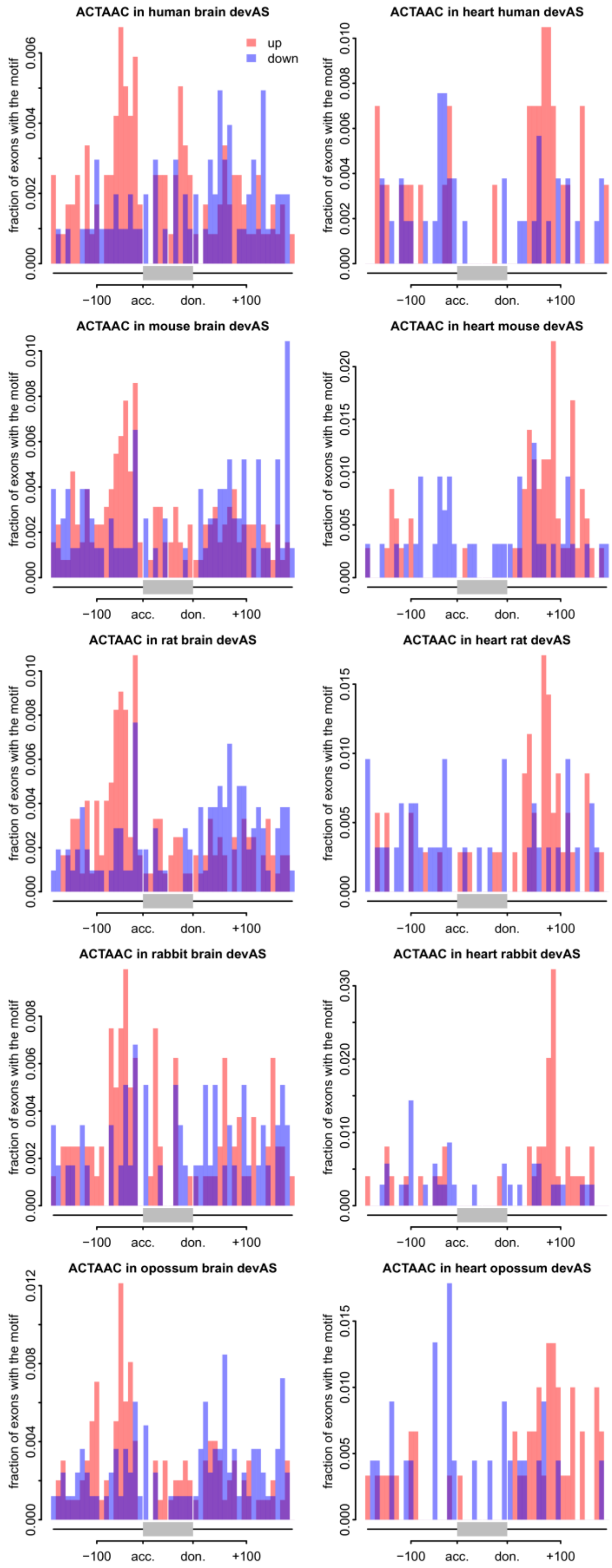
ACTAAC in human brain devAS
ACTAAC in heart human devAS
ACTAAC in mouse brain devAS
ACTAAC in heart mouse devAS
ACTAAC in rat brain devAS
ACTAAC in heart rat devAS
ACTAAC in rabbit brain devAS
ACTAAC in heart rabbit devAS
ACTAAC in opossum brain devAS
ACTAAC in heart opossum devAS
up
down
fraction of exons with the motif
−100
acc.
don.
+100

Supplementary Fig. 13 | Positional effect of QKI binding in brain and heart. Distribution of the ACTAAC hexamer in introns flanking brain (left) and heart (right) devAS cassette exons. This figure complements main Fig. 4g.

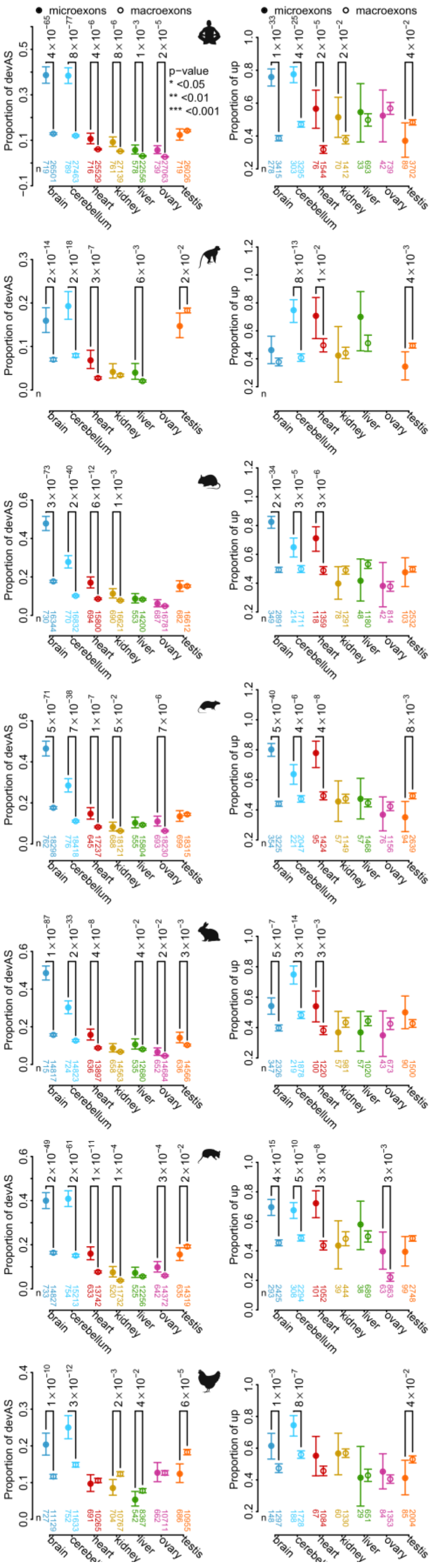
microexons
macroexons
Proportion of devAS
Proportion of up
p−value
* <0.05
** <0.01
*** <0.001
brain
cerebellum
heart
kidney
liver
ovary
testis

Supplementary Fig. 14 | Microexons in development. Left column: proportions of human microexons and macroexons with devAS among all cassette exons of the respective type. Right column: proportions of exons with increasing PSI ("up") among microexons and macroexons with devAS. Two-sided Fisher's exact tests were performed for statistical comparisons (*P < 0.05, **P < 0.01, ***P < 0.001). This figure complements main Fig. 5b, c. Dots show the mean values and error bars indicate the 95% confidence intervals based on binomial distributions. Species icons (except human) are from a previous study[18].

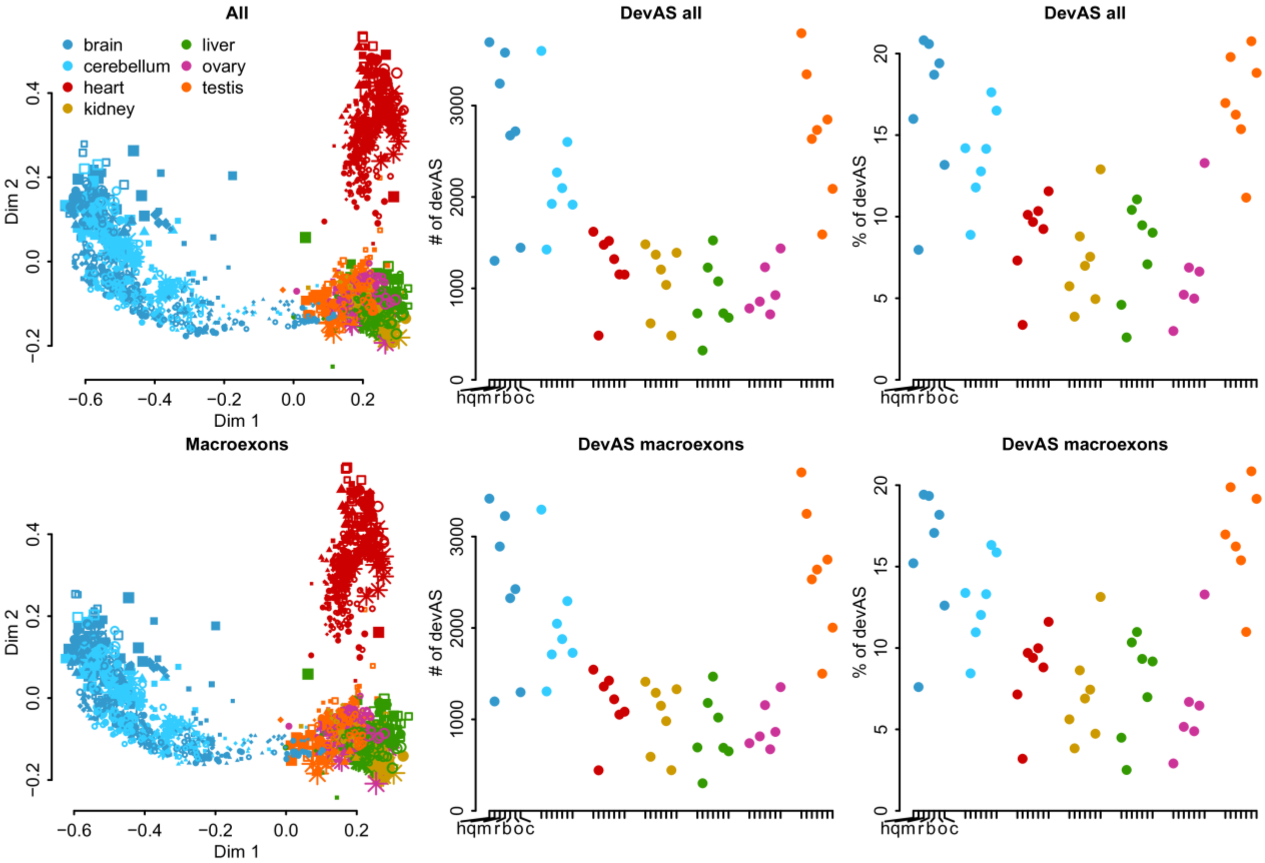

Supplementary Fig. 15 | DevAS patterns are reproduced in macroexon-restricted analyses. Left: MDS plots for all 1,890 samples based on pairwise distances (1 − *r*, Pearson correlation coefficient) between PSI values of all orthologous cassette exons (top, same as in main Fig. 2a) and for all orthologous cassette macroexons (bottom). **Middle:** Numbers of all devAS cassette exons (top) and macroexons (bottom) across organs and species. **Right:** Percentages of all devAS cassette exons (top) and macroexons (bottom) across organs and species. As these figures illustrate, most cassette exons are macroexons.
