# Supplementary material for: Alternative splicing during mammalian organ development
Source: Nat Genet. 2021 May 3;53(6):925–34. doi: 10.1038/s41588-021-00851-w (PMC8187152; doi:10.1038/s41588-021-00851-w)
Supplement: Supplementary file 2 — Reporting Summary [file 41588_2021_851_MOESM2_ESM.pdf]

nature research

Corresponding author(s): Pavel V. Mazin
Margarida Cardoso-Moreira
Henrik Kaessmann

Last updated by author(s): Mar 3, 2021

Reporting Summary

Nature Research wishes to improve the reproducibility of the work that we publish. This form provides structure for consistency and transparency in reporting. For further information on Nature Research policies, see our Editorial Policies and the Editorial Policy Checklist.

Statistics

For all statistical analyses, confirm that the following items are present in the figure legend, table legend, main text, or Methods section.

n/a	Confirmed	
☐	☒	The exact sample size (*n*) for each experimental group/condition, given as a discrete number and unit of measurement
☐	☒	A statement on whether measurements were taken from distinct samples or whether the same sample was measured repeatedly
☐	☒	The statistical test(s) used AND whether they are one- or two-sided<br>*Only common tests should be described solely by name; describe more complex techniques in the Methods section.*
☒	☐	A description of all covariates tested
☐	☒	A description of any assumptions or corrections, such as tests of normality and adjustment for multiple comparisons
☐	☒	A full description of the statistical parameters including central tendency (e.g. means) or other basic estimates (e.g. regression coefficient) AND variation (e.g. standard deviation) or associated estimates of uncertainty (e.g. confidence intervals)
☐	☒	For null hypothesis testing, the test statistic (e.g. F, t, r) with confidence intervals, effect sizes, degrees of freedom and P value noted<br>*Give P values as exact values whenever suitable.*
☒	☐	For Bayesian analysis, information on the choice of priors and Markov chain Monte Carlo settings
☒	☐	For hierarchical and complex designs, identification of the appropriate level for tests and full reporting of outcomes
☐	☒	Estimates of effect sizes (e.g. Cohen's d, Pearson's r), indicating how they were calculated

Our web collection on statistics for biologists contains articles on many of the points above.

Software and code

Policy information about availability of computer code

Data collection	No software was used
Data analysis	We used hisat2 (v2.1.0) to map the RNA-seq reads against the reference genomes. We generated pairwise whole genome alignments using LASTZ (v1.02) and several USCS tools according to genomewiki.ucsc.edu. The alignment files were manipulated using samtools (1.2). We assembled transcripts using StringTie (v1.3.3b). Cross-species mapping of sequence coordinates was performed using the htsjdk liftover library (v2.11.0-3). We used SAJR (v0.0) to analyze alternative splicing. We performed multidimensional scaling analysis (MDS) using the cmdscale function from R. We used Exon Ontology (http://fasterdb.ens-lyon.fr/ExonOntology/) to annotate human exons. We used BLASTN v2.9.0+ to align exons across species. All statistical analyses and plots were done in R (3.3.1) as implemented in Rstudio (1.0.136). Plots were created using the R basic graphics. Following R packages were used: GenomicAlignments (v1.24), reshape (v0.8.8), png (v0.1-7), ape (v5.3), seqinr (v3.6-1)

For manuscripts utilizing custom algorithms or software that are central to the research but not yet described in published literature, software must be made available to editors and reviewers. We strongly encourage code deposition in a community repository (e.g. GitHub). See the Nature Research guidelines for submitting code & software for further information.

Data

Policy information about availability of data

All manuscripts must include a data availability statement. This statement should provide the following information, where applicable:

- Accession codes, unique identifiers, or web links for publicly available datasets
- A list of figures that have associated raw data
- A description of any restrictions on data availability

All data are available in the main text, the supplementary materials, and/or our accompanying database (https://apps.kaessmannlab.org/alternative-splicing/), as well as other public databases: Ensembl (https://www.ensembl.org/index.html), Human Gene Mutation Database (http://www.hgmd.cf.ac.uk/ac/index.php), Exon Ontology database (http://fasterdb.ens-lyon.fr/ExonOntology/), CISBP-RNA (http://cisbp-rna.ccbr.utoronto.ca/index.php), and gnomAD (https://gnomad.broadinstitute.org/)

Field-specific reporting

Please select the one below that is the best fit for your research. If you are not sure, read the appropriate sections before making your selection.

☒ Life sciences ☐ Behavioural & social sciences ☐ Ecological, evolutionary & environmental sciences

For a reference copy of the document with all sections, see nature.com/documents/nr-reporting-summary-flat.pdf

Life sciences study design

All studies must disclose on these points even when the disclosure is negative.

Sample size	RNA-Seq data used in this study were taken from Cardoso-Moreira et al. paper (2019, doi: 10.1038/s41586-019-1338-5). All 1890 samples that passed filtering in original study were used. Dataset included samples for 7 major organs from early organogenesis to adulthood in human, rhesus macaque, mouse, rat, rabbit, opossum and chicken. The time points cover the most important periods of organ development and varied from 9 time points in chicken to 24 in human. For each individual analysis we used as many samples/ genes as possible. Precise sample sizes for each analysis are indicated in the Figure legends.
Data exclusions	No data was excluded
Replication	The dataset used in this study contains biological replicates for the stages and organs sampled in all species. Most samples have 4 biological replicates (2 males and 2 females) for somatic organs (2 for primates) and 2 replicates for the gonads. The analyses described in the manuscript jointly consider information from all available biological replicates. All attempts at replication were successful.
Randomization	This study did not perform any allocation into control or experimental groups and therefore sample randomization doesn't apply. All comparisons in this work are based on 3 biological variables, species, organ and developmental stage, thus random allocation to groups was not applicable.
Blinding	Blinding was not relevant to our study. Analyses required an understanding of the nature of the sample being analyzed (i.e. species, organ, developmental stage).

Reporting for specific materials, systems and methods

We require information from authors about some types of materials, experimental systems and methods used in many studies. Here, indicate whether each material, system or method listed is relevant to your study. If you are not sure if a list item applies to your research, read the appropriate section before selecting a response.

Materials & experimental systems

n/a	Involved in the study
☒	☐ Antibodies
☒	☐ Eukaryotic cell lines
☒	☐ Palaeontology and archaeology
☒	☐ Animals and other organisms
☒	☐ Human research participants
☒	☐ Clinical data
☒	☐ Dual use research of concern

Methods

n/a	Involved in the study
☒	☐ ChIP-seq
☒	☐ Flow cytometry
☒	☐ MRI-based neuroimaging
