# Supplementary material for: Alternative splicing during mammalian organ development
Source: Nat Genet. 2021 May 3;53(6):925–34. doi: 10.1038/s41588-021-00851-w (PMC8187152; doi:10.1038/s41588-021-00851-w)
Supplement: Supplementary file 3 — Peer Review Information [file 41588_2021_851_MOESM3_ESM.pdf]

natureresearch

Peer Review Information

Journal: Nature Genetics
Manuscript Title: Alternative splicing during mammalian organ development
Corresponding author name(s): Professor Henrik Kaessmann

Editorial Notes:

**Transferred manuscripts**	This manuscript has been previously reviewed at another journal that is not operating a transparent peer review scheme. This document only contains reviewer comments, rebuttal and decision letters for versions considered at Nature Genetics.

Reviewer Comments & Decisions:

Decision Letter, initial version:

Our ref: NG-AN55775-T

2nd Feb 2021

Dear Dr. Kaessmann,

Thank you for submitting your revised manuscript "Alternative splicing during mammalian organ development" (NG-AN55775-T). It has now been seen by the original referees and their comments are below. The reviewers find that the paper has improved in revision, and therefore we'll be happy in principle to publish it in Nature Genetics, pending minor revisions to comply with our editorial and formatting guidelines.

***Please note-
In order to proceed, we ask that you please send a word.doc version of the manuscript text, methods text, figure legend text and references. No figures or extended data figures need to be included. This will aid us in our formatting checks.
Can you please send this to me as soon as possible?

We are now performing detailed checks on your paper and will send you a checklist detailing our editorial and formatting requirements in about a week. Please do not upload the final materials and

make any revisions until you receive this additional information from us.

Thank you again for your interest in Nature Genetics Please do not hesitate to contact me if you have any questions.

Congratulations on the paper!

All the best,

Catherine

Catherine Potenski, PhD
Chief Editor
Nature Genetics
1 NY Plaza, 47th Fl.
New York, NY 10004
catherine.potenski@us.nature.com
https://orcid.org/0000-0002-4843-7071

Reviewer #1 (Remarks to the Author):

Note : This is my second revision of this manuscript (previously Referee #3).

The manuscript entitled “Alternative splicing during mammalian organ development”, by Mazin et al., presents a comparative analysis alternative splicing during development (devAS) in 7 species (6 mammals and chicken). The analysis is thorough and comprehensive, ranging from AS developmental patterns and their conservation, the apparition and evolution of new AS events, and associated genome structures (microexons, DNA motifs and transposable elements). It is the first AS analysis encompassing such a large dataset in development. The manuscript is clearly written. I think the manuscript is a very valuable contribution to the open debate on the functional importance of AS in mammalian genomes. This manuscript represents a huge work, the data is carefully analyzed and is a very useful resource for future work.

In my first revision, I only had a couple of requests, mainly to clarify and quantify some verbal assessment in the manuscript. The authors have answered these completely, in a very thorough and complete manner, and sometimes beyond my expectancies. I would like to thank them for this effort.

A number of figures and complementary analyses have been made, in order to answer the remarks of the 3 referees. I think the addition of the Extended Data Fig1 is very efficient to show the difference of developmental sampling across species, and was a very good idea. The authors also assessed, by subsampling analyses, how differences in developmental sampling might explain differences in the amount of detected AS and devAS events between species. They show convincingly that this is not the case (new Extended Data Fig. 4 & 14).

Regarding the question of tissue composition : I appreciate that it is not possible to deconvolve the

devAS patterns of the testis developmental bulk-tissue using cell type-specific RNA-seq data. The authors made it clear in their rebuttal and in the new text. They also acknowledge in the text, that “one important limitation in that the underlying bulk-tissue RNA-seq data do not generally allow us to assess the relative contributions of changes in cellular composition versus change”.

I’m also fully convinced by the answers to my other questions, and to the answer to the questions from the 2 other reviewers.

Reviewer #2 (Remarks to the Author):

The authors satisfactorily addressed most of my previous comments. It is acceptable for publication on Nature Genetics in my opinion and this work will be a great resource for the community.

Final Decision Letter:

In reply please quote: NG-AN55775R Kaessmann

19th Mar 2021

Dear Dr. Kaessmann,

I am delighted to say that your manuscript "Alternative splicing during mammalian organ development" has been accepted for publication in an upcoming issue of Nature Genetics.

Prior to setting your manuscript, we may make minor changes to enhance the lucidity of the text and with reference to our house style. We therefore ask that you examine the proofs most carefully to ensure that we have not inadvertently altered the sense of your text in any way.

Once your manuscript is typeset and you have completed the appropriate grant of rights, you will receive a link to your electronic proof via email with a request to make any corrections within 48 hours. If, when you receive your proof, you cannot meet this deadline, please inform us at rjsproduction@springernature.com immediately.

Your paper will be published online after we receive your corrections and will appear in print in the next available issue. You can find out your date of online publication by contacting the Nature Press Office (press@nature.com) after sending your e-proof corrections. Now is the time to inform your Public Relations or Press Office about your paper, as they might be interested in promoting its publication. This will allow them time to prepare an accurate and satisfactory press release. Include your manuscript tracking number (NG-AN55775R) and the name of the journal, which they will need when they contact our Press Office.

Before your paper is published online, we shall be distributing a press release to news organizations worldwide, which may very well include details of your work. We are happy for your institution or funding agency to prepare its own press release, but it must mention the embargo date and Nature Genetics. Our Press Office may contact you closer to the time of publication, but if you or your Press Office have any enquiries in the meantime, please contact press@nature.com.

Acceptance is conditional on the data in the manuscript not being published elsewhere, or announced in the print or electronic media, until the embargo/publication date. These restrictions are not intended to deter you from presenting your data at academic meetings and conferences, but any enquiries from the media about papers not yet scheduled for publication should be referred to us.

Nature Genetics is a Transformative open access journal. In approximately 10 business days you will receive an email with a link to choose the appropriate publishing options for your paper and our Author Services team will be in touch regarding any additional information that may be required.

Please note that Nature Research offers an immediate open access option only for papers that were first submitted after 1 January, 2021.

You will not receive your proofs until the publishing agreement has been received through our system.

If you have any questions about our publishing options, costs, Open Access requirements, or our legal forms, please contact ASJournals@springernature.com

In the event that you choose to publish under the subscription model, Nature Research allows authors to self-archive the accepted manuscript (the version post-peer review, but prior to copy-editing and typesetting) on their own personal website and/or in an institutional or funder repository where it can be made publicly accessible 6 months after first publication, in accordance with our self-archiving policy. Please review our self-archving policy for more information.

Several funders require deposition the accepted manuscript (AM) to PubMed Central or Europe PubMed Central. To enable compliance with these requirements, Nature Research therefore offers a free manuscript deposition service for original research papers supported by a number of PMC/EPMC participating funders. If you do not choose to publish immediate open access, we can deposit the accepted manuscript in PMC/Europe PMC on your behalf, if you authorise us to do so.

If you have posted a preprint on any preprint server, please ensure that the preprint details are updated with a publication reference, including the DOI and a URL to the published version of the article on the journal website.

To assist our authors in disseminating their research to the broader community, our SharedIt initiative provides you with a unique shareable link that will allow anyone (with or without a subscription) to read the published article. Recipients of the link with a subscription will also be able to download and print the PDF.

As soon as your article is published, you will receive an automated email with your shareable link.

natureresearch

You can now use a single sign-on for all your accounts, view the status of all your manuscript submissions and reviews, access usage statistics for your published articles and download a record of your refereeing activity for the Nature journals.

An online order form for reprints of your paper is available at https://www.nature.com/reprints/author-reprints.html. Please let your coauthors and your institutions' public affairs office know that they are also welcome to order reprints by this method.

If you have not already done so, we invite you to upload the step-by-step protocols used in this manuscript to the Protocols Exchange, part of our on-line web resource, natureprotocols.com. If you complete the upload by the time you receive your manuscript proofs, we can insert links in your article that lead directly to the protocol details. Your protocol will be made freely available upon publication of your paper. By participating in natureprotocols.com, you are enabling researchers to more readily reproduce or adapt the methodology you use. Natureprotocols.com is fully searchable, providing your protocols and paper with increased utility and visibility. Please submit your protocol to http://www.nature.com/protocolexchange/. After entering your nature.com username and password you will need to enter your manuscript number (NG-AN55775R). Further information can be found at http://www.natureprotocols.com.

Congratulations on the paper!

All the best,

Catherine

Catherine Potenski, PhD
Chief Editor
Nature Genetics
1 NY Plaza, 47th Fl.
New York, NY 10004
catherine.potenski@us.nature.com
https://orcid.org/0000-0002-4843-7071
